# *Nicotiana benthamiana* Kunitz peptidase inhibitor-like protein involved in chloroplast-to-nucleus regulatory pathway in plant-virus interaction

**DOI:** 10.3389/fpls.2022.1041867

**Published:** 2022-11-10

**Authors:** Natalia Ershova, Ekaterina Sheshukova, Kamila Kamarova, Evgenii Arifulin, Vadim Tashlitsky, Marina Serebryakova, Tatiana Komarova

**Affiliations:** ^1^ Vavilov Institute of General Genetics, Russian Academy of Sciences, Moscow, Russia; ^2^ Faculty of Bioengineering and Bioinformatics, Lomonosov Moscow State University, Moscow, Russia; ^3^ Belozersky Institute of Physico-Chemical Biology, Lomonosov Moscow State University, Moscow, Russia; ^4^ Chemistry Department, Lomonosov Moscow State University, Moscow, Russia

**Keywords:** plant-virus interaction, chloroplast retrograde signaling, carbon partitioning, potato virus X, plasmodesmata, Kunitz peptidase inhibitor-like protein (KPILP), organelles-nucleus-plasmodesmata signaling (ONPS)

## Abstract

Plant viruses use a variety of strategies to infect their host. During infection, viruses cause symptoms of varying severity, which are often associated with altered leaf pigmentation due to structural and functional damage to chloroplasts that are affected by viral proteins. Here we demonstrate that *Nicotiana benthamiana* Kunitz peptidase inhibitor-like protein (KPILP) gene is induced in response to potato virus X (PVX) infection. Using reverse genetic approach, we have demonstrated that *KPILP* downregulates expression of *LHCB1* and *LHCB2* genes of antenna light-harvesting complex proteins, *HEMA1* gene encoding glutamyl-tRNA reductase, which participates in tetrapyrrole biosynthesis, and *RBCS1A* gene encoding RuBisCO small subunit isoform involved in the antiviral immune response. Thus, *KPILP* is a regulator of chloroplast retrograde signaling system during developing PVX infection. Moreover, *KPILP* was demonstrated to affect carbon partitioning: reduced glucose levels during PVX infection were associated with *KPILP* upregulation. Another KPILP function is associated with plasmodesmata permeability control. Its ability to stimulate intercellular transport of reporter 2xGFP molecules indicates that KPILP is a positive plasmodesmata regulator. Moreover, natural KPILP glycosylation is indispensable for manifestation of this function. During PVX infection *KPILP* increased expression leads to the reduction of plasmodesmata callose deposition. These results could indicate that KPILP affects plasmodesmata permeability *via* callose-dependent mechanism. Thus, virus entering a cell and starting reproduction triggers *KPILP* expression, which leads to downregulation of nuclear-encoded chloroplast genes associated with retrograde signaling, reduction in photoassimilates accumulation and increase in intercellular transport, creating favorable conditions for reproduction and spread of viral infection.

## Introduction

Plants are constantly exposed to the environmental stress factors and respond to them by modulating the expression of an orchestra of numerous genes, thus adapting to the external stimuli. A coordinated and generalized plant response to adverse factors cannot do without intercellular communication *via* plasmodesmata (PD), which are unique plant cell structures that cross cell walls and connect the cytoplasm and endoplasmic reticulum (ER) of neighboring cells, forming a symplast (Lucas and Lee, 2004; Burch-Smith et al., 2011b; Burch-Smith and Zambryski, 2012; Patrick et al., 2013; Sager and Lee, 2014; Tilsner et al., 2016; Brunkard and Zambryski, 2017; Faulkner, 2018; Sager and Lee, 2018; Dorokhov et al., 2019; Sun et al., 2019). Intercellular transport is a very important process in plant life. Its regulation is complex and multilevel. Along with PD-localized factors that participate in PD structural and functional changes in response to different stimuli there is more and more evidence that PD are controlled by signals from other organelles, primarily from chloroplasts and mitochondria (Burch-Smith et al., 2011a; Burch-Smith and Zambryski, 2012; Azim and Burch-Smith, 2020; Ganusova et al., 2020). The coordinated functioning of intercellular compartments, accurate and timely transmission of signal molecules, and changes in the pattern of gene expression both at the transcriptional and translational levels are indispensable for the development, growth, and defense reactions of the whole plant.

More than one billion years ago, two independent endosymbiotic events occurred that led to the emergence of a eukaryotic cell with mitochondria and the ability to photosynthesize (Azim and Burch-Smith, 2020; Pfannschmidt et al., 2020). The plastids of the modern green land plants contain about 3000 proteins, more than 95% of which are encoded by a nuclear genome (Brunkard and Burch-Smith, 2018). Chloroplasts are the organelles that convert light energy and produce photoassimilates. But also they house many metabolic processes and play an important role in plant development, growth, and defense responses (Inaba and Ito-Inaba, 2010). Malfunctioning of the photosynthetic apparatus leads to an increase in ROS production and changes in the cell redox status, which in turn effects the pattern of expression of photosynthesis-associated nuclear genes (PhANGs) (Brunkard and Burch-Smith, 2018; Crawford et al., 2018). Many metabolic processes are carried out in plastids and their products determine the physiological status of the cell as they represent the components of the pathways for signal transduction from plastids to the nucleus. This form of signaling is designated as chloroplast retrograde signaling (CRS) (Burch-Smith and Zambryski, 2012). Currently, the CRS signals are classified into the following groups: biogenic signals that are generated by a plastid during its biogenesis and development; operational signals that are produced by a mature chloroplast in response to the environmental changes that affect its metabolism, these signals aimed to lead to stress adaptation; degradation signals are stress-induced signals from the plastids were degraded or destroyed (Crawford et al., 2018; Azim and Burch-Smith, 2020; Pfannschmidt et al., 2020). By modulating the expression of nuclear genes, CRS regulates not only the physiological status of the entire cell, but also affects intercellular communication. The elucidation of the relationship between *ISE2*, a chloroplast-resident RNA helicase that is involved in chloroplast RNA processing and translation, and PD permeability regulation became a starting point for the research of the chloroplast-PD interplay. Studies of *Arabidopsis thaliana ISE2* mutant of midtorpedo stage embryo have revealed the participation of chloroplasts in the regulation of cell-to-cell transport and PD formation (Kobayashi et al., 2007; Burch-Smith et al., 2011a; Bobik et al., 2017). This mutant is characterized by the increased intercellular traffic of 10-kD fluorescent dextran in the midtorpedo stage embryo and contains both simple and branched PD, while wild-type embryos at this stage of development contain only simple PD and limit dextran intercellular distribution (Kim et al., 2002; Kobayashi et al., 2007). Gene expression analysis of *A. thaliana ISE2* mutant embryos revealed changes in the expression of nuclear genes encoding plastid proteins participating in tetrapyrrole synthesis, Calvin–Benson cycle and photosynthetic electron transport chain components as well as genes with functions in cell wall biogenesis and modification (Burch-Smith et al., 2011a). Virus-induced gene silencing (VIGS) of *ISE2* in *Nicotiana benthamiana* leaves led to severe chlorosis, activation of the intercellular transport and secondary PD formation (Burch-Smith and Zambryski, 2010; Burch-Smith et al., 2011a). The interconnection between organelles’ functioning, nuclear gene expression and regulation of PD biogenesis and function underlies the hypothesis of organelle-nucleus-PD signaling (ONPS). It suggests that chloroplasts affecting carbon partitioning (carbon metabolism and translocation of the photoassimilates to the growing point) are the key regulators of all life processes and defense reactions (Burch-Smith et al., 2011a; Azim and Burch-Smith, 2020).

Plant viruses exploit the host plant cell using its resources. Viral infection leads to the depletion of the plant cell, since this process is extremely energy-consuming. Efficient viral propagation and systemic spread requires intracellular reproduction, intercellular trafficking *via* PD and long-distance transport *via* vasculature. To succeed, the virus “follows” certain strategies of the host exploitation. Viral infection often leads to the structural modification of cellular components especially membranous compartments. Virus-induced vesicular structures originating from nucleus, ER, peroxisomes, mitochondria or chloroplasts are used for viral replication complexes formation and/or transport (Laliberté and Sanfaçon, 2010). Viral proteins enter these compartments and take control of the organelles’ functioning, modulate the expression of nuclear genes, suppress antiviral immunity to create favorable conditions for reproduction and spread using components and resources of the host cell (Caplan et al., 2008; Zhao et al., 2016; Bhattacharyya and Chakraborty, 2018). Many viruses have chloroplast-targeting proteins encoded in their genome and/or use host cell proteins to reach the chloroplast (Qiao et al., 2009; Li et al., 2016; Bhattacharyya and Chakraborty, 2018; Souza et al., 2019). Exploitation of the cellular membrane compartments is advantageous for viral propagation: chloroplasts isolated by a double membrane from the intracellular environment as well as virus-induced membrane structures (viroplasms) can serve as a perfect site for formation of viral replication complexes (VRC) protecting viral RNA from the plant cell silencing machinery that functions in the cytoplasm (Tabler and Tsagris, 2004; Laliberté and Sanfaçon, 2010; Bhattacharyya and Chakraborty, 2018). Symptoms of a viral infection are often associated with chlorosis, mottle or mosaic of infected leaves are a consequence of structural and functional changes of chloroplasts caused by viral proteins directly attacking chloroplasts and/or interacting with chloroplast proteins (Reinero and Beachy, 1989; Qiao et al., 2009; Bhat et al., 2013; Li et al., 2016; Budziszewska and Obrępalska-Stęplowska, 2018). Viral infection leads to suppression of the chloroplast photosynthetic functions and activation of genes related to defense reactions including jasmonate and salycilate pathways (Reinero and Beachy, 1989; Bilgin et al., 2010; Souza et al., 2019).

Plant viruses induce numerous modifications in host gene expression pattern. Despite the accumulated data on plant-virus interactions and transcriptomic changes in response to viral infection, the mechanisms by which viruses affect host gene expression patterns are still not fully understood. Besides genes encoding components associated with defense reactions virus invasion leads to upregulation of the multiple genes the function of which is to be elucidated and the role for the viral propagation is unknown. Among these genes is recently identified *Nicotiana benthamiana* stress-induced gene encoding Kunitz protease inhibitor-like protein (KPILP) with unknown function (Sheshukova et al., 2017). Its expression is very low in intact leaves and increases in response to tobacco mosaic virus (TMV) infection. *KPILP* mRNA level correlates with TMV accumulation and chloroplast dysfunction. Moreover, this gene is highly expressed in roots and is activated in leaves in response to prolonged darkness (Sheshukova et al., 2017; Sheshukova et al., 2018). Despite KPILP contains a KPI domain it was shown to have no protease inhibitor activity that could be explained by the lack of amino acid residues that are indispensable for this functional activity (Sheshukova et al., 2017).

Here we study *KPILP* function and its role in the viral infection. We showed that potato virus X (PVX) systemic infection induces *KPILP* expression in *Nicotiana benthamiana* plants up to 9-fold compared to the intact plant. PVX infection activates CRS pathway as was demonstrated by the analysis of photosynthesis-associated nuclear-encoded genes (PhANGs) and CRS marker genes expression that is downregulated. Due to general photosynthesis suppression the level of glucose decreases approximately 4-fold in source and 2-fold in sink leaves compared to the intact plant. To understand *KPILP* role in PVX–chloroplast interaction we used virus-induced gene silencing (VIGS) approach for *KPILP* knockdown and demonstrated that *KPILP* suppression during PVX infection prevents CRS marker genes downregulation and suppression of photosynthetic function. Thereby, photoassimilates level in sink leaves remains the same as in the intact plant. As for source leaves, glucose content is 3-fold higher in leaves with *KPILP* knockdown compared to PVX-infected leaves with upregulated *KPILP*. Thus, *KPILP* participates in CRS and glucose metabolism during PVX infection. Moreover, we have shown that PVX-induced *KPILP* expression is associated with downregulation of PD callose depositions and increased intercellular transport of macromolecules.

## Materials and methods

### Plant growth conditions

Wild type *Nicotiana benthamiana* plants as well as ΔXTFT (Strasser et al., 2008) transgenic *N. benthamiana* plants were grown in soil in a controlled environment chamber under a 16 h/8 h day/night cycle.

### Plasmid constructs

For silencing of endogenous *KPILP* gene, we used a PVX genome-based expression vector. 183 nt *KPILP* fragment (from 70 to 253 nt of KPILP coding region) was amplified using F2/R2 pair of primers. PCR product was digested with NruI-SalI enzymes and inserted in pPVX201 vector (Baulcombe et al., 1995) under control of duplicated CP promoter. To obtain pPVX(frKPILP) vector a fragment from the resulting subclone was transferred into PVX-BIN19 (Komarova et al., 2006) *via* AvrII/SalI sites.

The set of plasmids encoding KPILP N-mutants was obtained *via* the following cloning steps. The first step was overlap PCR with two (four in the case of the triple mutant) pairs of primers: for 35S-KPILP(N60A) - F1, R1, F8 and R8; for 35S-KPILP(N86A) - F1, R1, F9 and R9; for 35S-KPILP(N136mut) - F1, R1, F10 and R10. 35S-NbKPILP(ACG) plasmid (Sheshukova et al., 2017) was used as a template. The second step was digesting the obtained PCR product with Acc65I and SalI restriction enzymes, the recognition sites of which were introduced in the primers. The last step was the ligation of the fragment with “sticky ends” into pCambia-35S (Sheshukova et al., 2017) that was digested with the same enzymes.

The plasmid 35S-2xGFP//35S-RFP:NLS was constructed in two steps. First, the expression cassette including the 35S promoter and gene encoding translational fusion between RFP and simian virus 40 T-antigen NLS was assembled in pUC-based plasmid pFF19 (Timmermans et al., 1990). Second, the assembled cassette was transferred to pBin19 containing 35S-2xGFP-cassette (Dorokhov et al., 2012) *via* NheI and ApaI sites.

The oligonucleotide sequences are listed in **Table S1**.

### Agroinjection experiments

*Agrobacterium tumefaciens* strain GV3101 was transformed with individual binary constructs and grown at 28°C in LB medium supplemented with 50 mg/l rifampicin, 25 mg/l gentamycin and 50 mg/l carbenicillin/kanamycin. *Agrobacterium* overnight culture was diluted in agrobuffer containing 10 mM MES (pH 5.5) and 10 mM MgSO_4_ and adjusted to a final OD_600_ of 0.1. In experiments with 2×GFP, the final OD_600_ for 35S-2×GFP//35S-RFP:NLS was 0.005. Agrobacterium containing pPVX or pPVX(frKPILP) was diluted to OD_600_ of 0.01. Agroinfiltration was performed on almost fully expanded *N. benthamiana* leaves that were still attached to the intact plant. A bacterial suspension was infiltrated into the leaf tissue using a 2-ml syringe, after which the plants were incubated under greenhouse conditions.

### PVX inoculation

*N. benthamiana* plants were inoculated by pPVX or pPVX(frKPILP) *via* agroinfiltration of the lower leaves and in 10-14 days the systemic PVX infection was detected in the upper leaves.

### GFP and RFP imaging

GFP fluorescence was detected using an AxioVert 200M microscope (Carl Zeiss, Germany) equipped with an AxioCam MRc digital camera. The excitation and detection wavelengths for GFP were 487 nm and 525 nm, respectively; the excitation and emission wavelengths for RFP were 561 nm and 625 nm, respectively. The lower epidermal cells from injected leaves were analyzed 20-24 h after infiltration experiments with 2×GFP. Not less than 200 cell clusters per one infiltration area was analyzed and not less than 3 biological repeats per experiment. Three to four experiments were performed.

### Callose staining and quantification

To visualize PD-located callose, PVX-infected *N. benthamiana* leaves with up- or downregulated *KPILP*, were infiltrated with aniline blue solution (0.1% aniline blue (Sigma Aldrich) in 0.01 M K_3_PO_4_ at pH 12). Then the leaves were incubated in the dark at room temperature for 15 minutes before imaging using a Nikon C2 laser scanning confocal microscope. The excitation and emission wavelengths for aniline-blue-stained callose were 403 nm and 447 nm, respectively. Quantification of callose fluorescence was performed as described by Zavaliev and Epel (2015).

### Protein extracts preparation

Total soluble protein from PVX-infected leaves was extracted by homogenization of leaf fragment in 1× PBS buffer followed by centrifugation (16,000 × *g* 10 min) for extract clarification.

The samples from 35S-KPILP-agroinfiltrated leaves were harvested 3 dpi and KPILP-enriched protein extracts for PNGase F treatment were prepared as follows. 0.5 g of leaf material were ground to powder in liquid nitrogen followed by the addition of 3 volumes of extraction buffer (100 mM Tris, pH 8.0, 0.4 M sucrose, 10 mM KCl, 5 mM MgCl_2_, 10 mM β-mercaptoethanol and 0,1 mM PMSF). The obtained slurry was filtered through a double-layered Miracloth (Millipore/Merck, USA). The material retained on the filter was collected and washed (30-60 min incubation followed by centrifugation at 1000 × *g*) 5-8 times with the extraction buffer supplemented with 0.1% Triton X-100. When the pellet lost its green color it was washed with the extraction buffer without Triton X-100 and resuspended in one volume of 100 mM sodium-phosphate buffer (pH 7.5).

### PNGase F treatment

The KPILP-enriched protein fraction was obtained from the agroinfiltrated *N. benthamiana* plants. PNGase F (New England Biolabs, USA) treatment was performed according to the manufacturer’s protocol under denaturing conditions.

### Western blot analysis

Aliquots from protein extracts were analyzed by SDS-polyacrylamide gel electrophoresis and blotted onto polyvinylidene difluoride membranes (GE Healthcare, USA). For KPILP detection, the membranes were probed with polyclonal antibodies against KPILP-6His raised in rabbit (Almabion, Russia). Anti-rabbit antibodies conjugated with horseradish peroxidase (Rockland Immunochemicals, USA) were used as secondary antibodies. The bands were visualized using a chemiluminescence ECL kit (GE Healthcare, USA) and X-ray film.

### Measurement of glucose content in plant tissues

Glucose content was assessed as described earlier (Sheshukova et al., 2017). Briefly, dried leaf samples (30 mg) were hydrolyzed with 1 ml of 1M hydrochloric acid (100°C for 2.5 hours). The resulting solution was centrifuged for 10 minutes at 14,000xg. 0.5 ml of the supernatant was diluted with 1.5 ml of water and loaded to a reversed-phase concentrating cartridge (Diasorb C16), the first 1.8 ml was discarded and the next 0.2 ml was collected. Glucosamine solution (1 g/L) was used as an internal standard for each sample. Mixture of equal volume (20 μl) of standard and sample was evaporated on a SpeedVac vacuum centrifugal evaporator with heating. 20 μl 0.5 M solution of PMP (1-phenyl-3-methyl-5-pyrazolone) in methanol and 20 μl of 0.3 M KOH were added to a dried sample and incubated at 70°C for 2 hours. The sample was neutralized by the addition of 20 μl of 0.3 M hydrochloric acid and the excess of the PMP reagent was extracted twice with benzene. The residue was evaporated on a SpeedVac with heating and dissolved in acetonitrile/water (1:9). The test mixture and analytical samples were analyzed by reversed-phase HPLC in a gradient mode on a Luna C18 (2) 4.6x250 mm (5 μm) column with the mobile phase A – water, B –acetonitrile and D – 100 mM potassium hydrogen phosphate in water (pH 9.12) at a flow rate of 1 ml/min, a temperature of 25°C and with UV detection at 260 nm using a gradient chromatograph Agilent 1100 with PDA detector. Collection and processing of chromatograms was carried out with ChemStation (Agilent) and AutoChrom1200 (ACDlabs) programs.

### Quantitative real-time PCR analysis of transcript concentrations

Total RNA was extracted from plant tissues using the TriReagent (MRC, USA) according to the manufacturer’s instructions. The synthesis of first strand cDNA, followed by real-time qPCR was performed as described in (Dorokhov et al., 2012). Briefly, 0.1 mg of random hexamers and 0.1 mg of oligo-dT primer were added to 2 mg of total RNA to obtain cDNA by reverse transcription performed using Superscript IV reverse transcriptase (Invitrogen, Waltham, MA, USA) according to the manufacturer’s protocol. Real-time quantitative PCR was carried out using the iCycler iQ real-time PCR detection system (Bio-Rad, Hercules, CA, USA). Reference genes were detected using the primers to 18S rRNA gene and protein phosphatase 2A gene (PP2A); target genes were detected using sequence-specific primers and Eva Green master mix (Syntol, Russia) according to the manufacturer’s instructions. Primers used for qRT-PCR are listed in the **Table S2**. Each sample was run in triplicate, and a nontemplate control was added to each run. A minimum of five biological replicates were performed. The qRT-PCR results were evaluated using the Pfaffl algorithm (Pfaffl, 2001).

### Statistical analysis

The data was first analyzed for the normality of distribution (Shapiro-Wilk test) and homogeneity of variances (Levene test). The set of values satisfied the Shapiro-Wilk test for the normality of distribution were analyzed by one-way ANOVA. The significance of difference between groups were further assessed using Tukey’s honestly significant difference (HSD) test at *p* < 0.05 level. For the data that did not satisfy normality of distribution, Kruskal-Wallis non-parametric test was applied. The pairwise comparisons between groups was then performed by *post-hoc* Dunn’s test with Bonferroni correction. In all histograms, y-axis error bars represent the standard error of the mean values.

## Results

### PVX infection leads to KPILP mRNA accumulation

In intact mature *N. benthamiana* leaves *KPILP* mRNA accumulation is suppressed due to the expression of a nested alternative open reading frame. But the reproduction of viral vector crTMV:GFP based on the genome of crTMV induces a significant increase in the level of *KPILP* mRNA. Systemic TMV infection of *N. tabacum* was also characterized by intensive *KPILP* accumulation, especially in the yellow-green mosaic areas enriched with TMV particles (Sheshukova et al., 2017).

**Figure 1:**
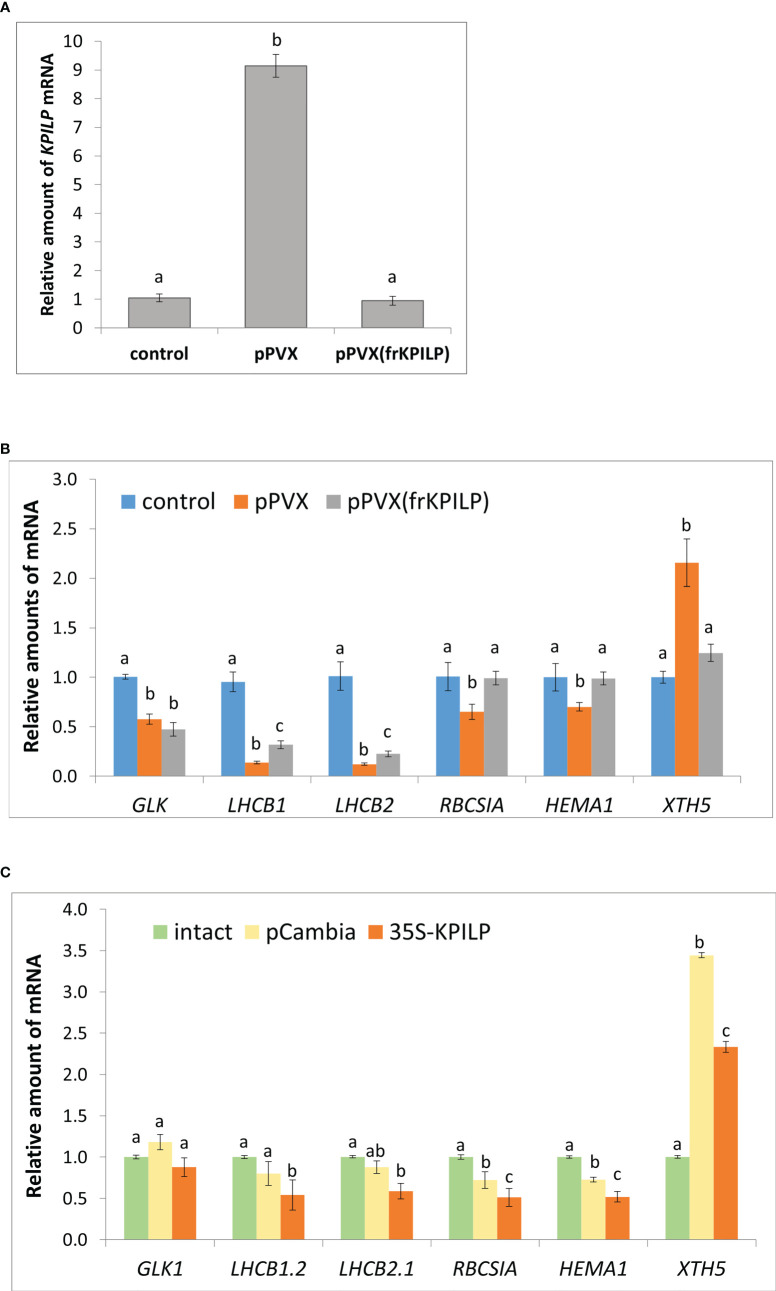
*KPILP* is involved in CRS regulatory hub. **(A)** Relative amount of *KPILP* mRNA in leaves with systemic PVX infection (pPVX or pPVX(frKPILP)) as determined by qPCR. Mean values and standard error are presented. The data was analyzed using ANOVA. Bars without shared letters indicate significant differences according to Tukey’s HSD at p<0.05. **(B)** Relative amount of CRS marker genes mRNA in response to PVX infection and *KPILP* downregulation as determined by qPCR. Mean values and standard errors are presented. The level of mRNA accumulation for each gene in a control plant was taken as 1. The data was analyzed using Kruskal-Wallis test. Bars with different letter indicate significant difference according to *post-hoc* Dunn’s test at p<0.05 while bars with shared letter are not significantly different. **(C)** Relative amount of CRS marker genes mRNA in leaves 3 days after agroinfiltration with “empty” pCambia1300 or 35S-KPILP as determined by qPCR. Mean values and standard errors are presented. The level of mRNA accumulation for each gene in an intact plant was taken as 1. Bars with different letter indicate significant difference at p<0.05 (ANOVA, Tukey’s HSD), while bars with shared letter are not significantly different.

To understand whether *KPILP* induction is a part of a general antiviral response rather than only a reaction to tobamoviral infection we inoculated *N. benthamiana* plants with potato virus X (PVX) using a viral vector pPVX [PVX-BIN19 (Komarova et al., 2006)] encoding PVX infectious copy (**Figure S1A**, left). We observed symptoms of the systemic infection in the upper leaves of the plants 10-14 days after agroinfiltration (**Figure S1B**, left). We assessed *KPILP* mRNA content in systemic leaves using quantitative real-time PCR (qRT-PCR) approach and revealed 9-fold increase in *KPILP* expression level in plants infected with pPVX compared to the intact plants (**Figure 1A**).

Thus, *KPILP* mRNA accumulation is elevated in response to PVX infection.

### KPILP regulates nuclear encoded chloroplasts genes expression in CRS pathway

Earlier it was demonstrated, that *KPILP* mRNA level is high in roots and upregulated in leaves after prolonged plant incubation in the dark (Sheshukova et al., 2017). Together with the fact that photosynthesis is suppressed during productive viral infection these allows to suggest that *KPILP* could be associated with chloroplast functioning or photosynthesis.

To elucidate *KPILP* role in virus-host interaction we performed *KPILP* knockdown in *N. benthamiana* plant using PVX-based vector for virus-induced gene silencing (VIGS) approach (Burton et al., 2000; Shimizu et al., 2009). We constructed a pPVX(frKPILP) viral vector that contained a 183-nt *KPILP* fragment under control of a duplicated CP subgenomic promoter (**Figure S1A**, right). *N. benthamiana* leaves were inoculated by pPVX(frKPILP) *via* agroinfiltration and in 10-14 days the systemic PVX infection was detected in the upper leaves (**Figure S1B**, right). The level of viral reproduction in plants with pPVX(frKPILP) or pPVX systemic infection was not significantly different as was demonstrated by assessment of PVX genomic RNA levels (**Figure S2A**) but coat protein accumulation was slightly lower in pPVX(frKPILP) plants (**Figure S2B**). We analyzed *KPILP* mRNA accumulation level in upper leaves of intact plants and plants inoculated with pPVX(frKPILP) or pPVX and demonstrated that *KPILP* was downregulated by 90% in silenced plants compared to pPVX-infected plants and decreased to the levels characteristic of the intact plant of the same age (**Figure 1A**). Thus, we developed a model system that allows to compare effects of *KPILP* up- and downregulation during PVX infection.

We hypothesize that *KPILP* is involved in the regulatory pathways for the transmission of signals from chloroplasts to the nucleus. To test this assumption, we assessed the expression of the listed below genes which are responsive to CRS, i.e. the changes of their expression indicate the launch of the chloroplast retrograde signaling. *GOLDEN2-LIKE1* (*GLK1*) encodes transcriptional factor essential for normal chloroplast development (Fitter et al., 2002). *GLK1* expression correlates with photosynthesis-associated genes and it is suppressed when functioning of chloroplasts is impaired (Kakizaki et al., 2009). *LHCB 1* and *2* encode the antennae proteins of the photosystem-associated light-harvesting complexes (LHC); *RBCS1A* encodes one of the isoforms of rubisco small subunit that is involved in antiviral plant immune response (Bhat et al., 2013). Changes in *LHCB 1, 2* and *RBCS1A* expression coordinates retrograde signals that define physiological status of chloroplasts (Susek et al., 1993; Burch-Smith et al., 2011a). *HEMA1* is another nuclear gene encoding chloroplast protein, *viz* glutamyl-tRNA reductase that mediates the first step of tetrapyrrole biosynthesis (Schmied et al., 2011).

The qRT-PCR analysis of the corresponding mRNA showed that in pPVX-infected plant where *KPILP* is activated the expression of all these genes is suppressed compared to the intact plant. While in pPVX(frKPILP)-infected plants with suppressed *KPILP*, *LHCB1* and *LHCB2* are slightly downregulated compared to the intact plant (**Figure 1B**). Nevertheless, *LHCB1* and *LHCB2* levels are significantly higher in *KPILP*-silenced leaves compared to pPVX-infected. Moreover, in *KPILP*-silenced plants PVX infection does not lead to *RBCS1A* and *HEMA1* downregulation: their expression remains at levels characteristic of the intact plants.

In addition, we analyzed the expression of *XTH5* gene that belongs to *XTH* gene family encoding xyloglucan endotransglucosylase/hydrolase. *XTH5* gene is playing a vital role in response to specific biotic or abiotic stresses and likely participates in xyloglucan remodeling and turnover during primary cell wall formation (Yokoyama and Nishitani, 2001; Ishida and Yokoyama, 2022). *XTH5* expression is regulated by ABSCISIC ACID INSENSITIVE4 (ABI4) and ELONGATED HYPOCOTYL5 (HY5) transcriptional factors that are involved in chloroplast-nucleus regulatory hub and plant development (Xu et al., 2016). We have demonstrated that *KPILP* mRNA level elevated in response to PVX infection correlates with *XTH5* expression while in *KPILP*-silenced plants *XTH5* expression is not affected despite they are also infected with PVX (**Figure 1B**).

To confirm that *KPILP* affects CRS marker genes expression we agroinfiltrated *N. benthamiana* leaves with 35S-KPILP to achieve increased levels of *KPILP* mRNA (**Figure S3**) and analyzed abovementioned genes expression. The results of qPCR analysis indicate that 35S-directed *KPILP* overexpression leads to the suppression of *LHCB 1, 2*, *RBCS1A* and *HEMA1* mRNA accumulation and stimulation of *XTH5* expression (**Figure 1C**). Noteworthy, agroinfiltration with a control “empty” binary vector pCambia1300 *per se* leads to the increase of *KPILP* mRNA level (**Figure S3**) inducing some changes in CRS genes expression (**Figure 1C**). However, most of these fluctuations are not statistically significant comparing to the intact plant while plasmid-directed *KPILP* overexpression launched more pronounced changes. Therefore, both viral-induced endogenous *KPILP* and transiently expressed from the plasmid affect the CRS marker genes in a similar way.

Together these results indicate that during PVX infection *KPILP* participates in operational retrograde signaling, negative regulation of *LHCB1* and *LHCB2*, *RBCS1A* and *HEMA1* genes expression and is associated with increased *XTH5* level.

### KPILP is necessary for maturation of the PsbQ subunit of oxygen-evolving complex during PVX infection

**Figure 2:**
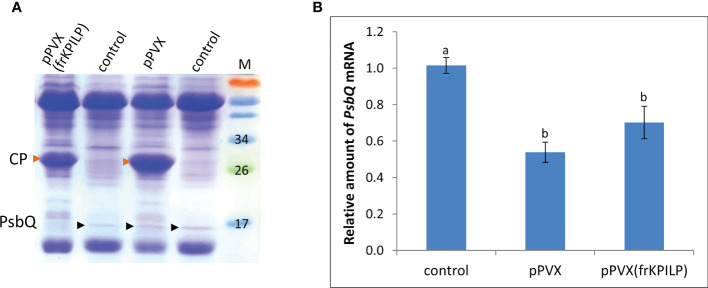
KPILP downregulation in PVX-infected *N. benthamiana* plants leads to decreased accumulation of PsbQ. **(A)** Analysis of total soluble protein from leaves with systemic infection induced by pPVX or pPVX(frKPILP) and control intact plants in polyacrylamide gel followed by Coomassie staining. Bands corresponding to PVX CP are indicated with red arrowheads, bands corresponding to PsbQ 16kDa protein are indicated with black arrowheads. M, protein weight markers. **(B)** Relative amount of *PsbQ* mRNA in the intact and PVX-infected plants as determined by qPCR. Mean values and standard errors are presented. The level of mRNA accumulation for control was taken as 1. Difference between control and samples from PVX-infected plants is significant at p<0.05 (ANOVA, Tukey’s HSD test). Bars without shared letters indicate significant differences. The difference between pPVX and pPVX(frKPILP) is significant at p<0.09.

We analyzed total soluble protein of PVX-infected *N. benthamiana* leaves with upregulated or silenced *KPILP*. In protein extracts from systemic leaves of pPVX-inoculated plant and leaves from intact plant we revealed a protein band that was absent in the samples from pPVX(frKPILP)-infected plants (**Figure 2**). Mass-spectroscopy analysis of the tryptic peptides obtained from this band indicated that it corresponded to the chloroplast oxygen-evolving protein 16 kDa subunit (PsbQ) (Uniprot Q5EFR5). PsbQ plays an important role in the luminal oxygen-evolving activity of photosystem II from higher plants and green algae. However, the analysis of *PsbQ* mRNA level in three studied variants of plants (**Figure 2**) showed that *PsbQ* expression is downregulated both in pPVX- and pPVX(frKPILP)-infected plants in the same extent compared to the intact plant. This indicates that the absence of the detectable amounts of PsbQ on the Coomassie-stained gel in pPVX(frKPILP)-infected plants is not solely a result of mRNA level decrease. We suggested that in *KPILP*-silenced PVX-infected plants PsbQ synthesis, maturation or targeting are impaired and functional PsbQ 16 kDa protein does not reach chloroplasts and/or degraded.

### KPILP regulates photoassimilates accumulation during PVX infection

**Figure 3:**
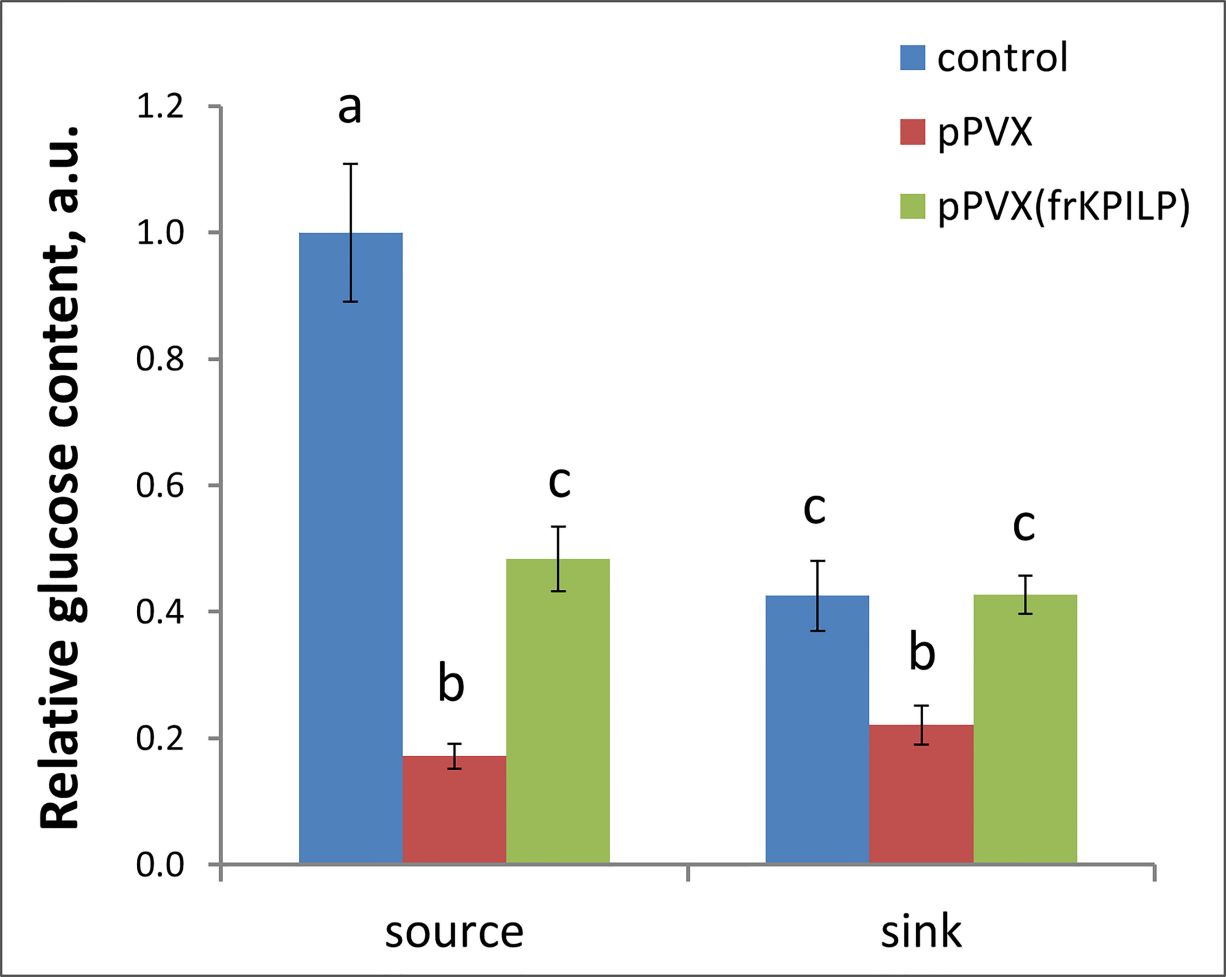
Relative glucose content in source leaves and sink tissues *of N. benthamiana* plants with pPVX or pPVX(frKPILP) systemic infection. The level of glucose in source leaves of control plants was taken as 1. Mean values and standard errors are presented. Bars without shared letters indicate significant differences at p<0.05 (ANOVA, Tukey’s HSD).

Photoassimilates are the product of photosynthesis, and their physiological distribution throughout the whole plant is necessary for proper growth, maturation, and reproduction. Viral infection negatively affects photosynthesis and leads to the exhaustion of the resources. We assessed the glucose level in sink and source leaves of the intact, pPVX- or pPVX(frKPILP)- infected plants. Source leaves are the mature leaves that produce the main volume of photoassimilates serving as a nutrients supply for the whole plant. The fraction of sink tissues included apical parts of the plant with young leaves less than 1 cm in length, flowers and flower buds. In both pPVX- and pPVX(frKPILP)-infected systemic source leaves we observed decrease in glucose level compared to the intact plants: 6-fold and 2-fold, respectively (**Figure 3**). This indicates the suppression of photosynthesis and carbon metabolism caused by viral infection. However, in sink tissues of the *KPILP*-silenced plants glucose level was the same as in intact plants and in pPVX-infected plants where *KPILP* is upregulated glucose content decreased 2-fold (**Figure 3**).

These results allow us to suggest that *KPILP* participates in carbon metabolism and partitioning during PVX infection and reduced glucose levels are associated with *KPILP* virus-induced upregulation.

### KPILP regulates callose deposition

**Figure 4:**
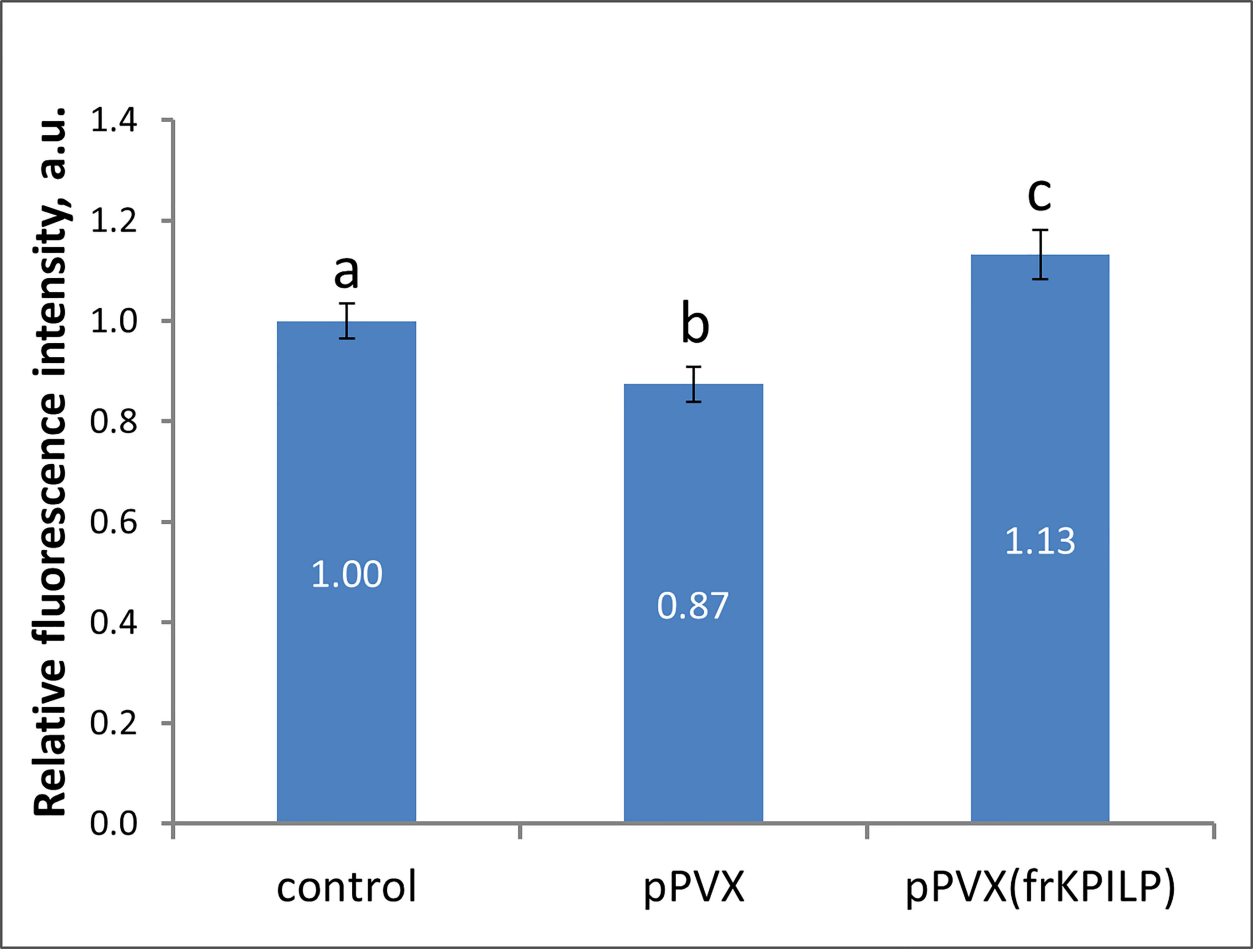
*KPILP* affects callose deposition around PD. Relative callose levels at PD as estimated by measurement of aniline blue-stained callose fluorescence intensity in *N. benthamiana* plants with PVX systemic infection and *KPILP* downregulation compared to the intact plants. The level callose in control plants was taken as 1. Mean values in arbitrary units (a.u.) and standard errors are presented. Bars without shared letters indicate significant differences at p<0.05 (ANOVA, Tukey’s HSD).

Biologically, being a virus-induced gene that plays a role in CRS and carbon partitioning, *KPILP* according to ONPS hypothesis could affect intercellular transport that changes in response to different stresses. One of the PD regulation mechanisms is modulation of callose depositions at PD. Increase in callose deposition induces the reduction of PD aperture and callose degradation results in PD dilation and activation of intercellular transport. Plant viruses could affect PD permeability to mediate cell-to-cell spread of the infection exploiting cellular factors. To assess the effect of *KPILP* overexpression on callose-mediated PD permeability regulation we analyzed PD-deposited callose in systemic leaves of pPVX- and pPVX(frKPILP)-infected *N. benthamiana* plants. Callose was stained with aniline blue (**Figure S4**) (Zavaliev and Epel, 2015) and quantified (**Figure 4**). The level of callose depositions in pPVX-infected plants with upregulated *KPILP* was reduced by 13% compared to the intact plants while leaves with *KPILP* knockdown demonstrated a 13% increase in fluorescence intensity of stained PD callose. Noteworthy, the number of the fluorescent dots and the mean area of them do not differ among analyzed samples indicating that neither plasmodesmata density nor their morphology significantly changed compared to the control plants. These results show that *KPILP* is associated with decreased callose levels during viral infection.

### KPILP stimulates intercellular transport of macromolecules

**Figure 5:**
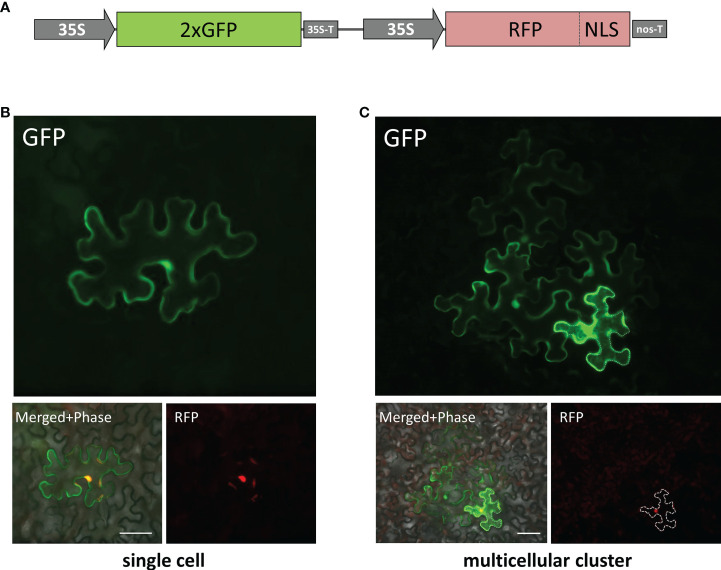
Visualization of 35S-2×GFP//35S-RFP:NLS expression and 2×GFP intercellular movement in epidermal cells of *N. benthamiana* leaves. **(A)** Schematic representation of 35S-2×GFP//35S-RFP:NLS construct containing two expression cassettes. Single cell **(B)** and multicellular cluster **(C)** in leaves agroinfiltrated with 2×GFP//35S-RFP:NLS supplemented with “empty vector” pBIN **(B)** or with 35S-KPILP **(C)**. The primary infected cell nucleus is indicated by the fluorescent signal from RFP and this cell outline is marked with a dashed line. Scale bar, 50 µm.

To evaluate cell-to-cell communication in leaves with elevated *KPILP* expression, we used a reporter macromolecule containing a fusion of two copies of green fluorescent protein (2×GFP) (54 kDa), as PD from intact mature source leaves are not permeable to proteins larger than 47 kDa (Crawford and Zambryski, 2000). To obtain individual cells or cell clusters expressing 2×GFP, we diluted an agrobacterial suspension to deliver the 35S-based 2×GFP-encoding plasmid to individual cells located distantly from each other. Although the primary transformed cell is clearly distinguishable by the decreasing fluorescence intensity from the cells to which 2xGFP moved (Burch-Smith and Zambryski, 2010), we created a vector, 35S-2xGFP//35S-RFP:NLS, containing two expression cassettes to mark each primary transformed cell nucleus with RFP fused to the nuclear localization signal (NLS) (**Figure 5A**) and used it for further experiments. Mature *N. benthamiana* leaves were examined using fluorescent light microscopy 20-24 h after joint agro-transformation with the 35S-2xGFP//35S-RFP:NLS and 35S-KPILP. Counting the number of epidermal cells surrounding the initial transformed cell that displayed fluorescence provides a quantitative estimation of 2×GFP movement (Burch-Smith and Zambryski, 2010; Dorokhov et al., 2012). When the PD were “closed”, 2×GFP was mainly detected in single cells (**Figure 5B**). While fluorescent signals were distributed in clusters containing 2, 3 or more cells (**Figure 5C**) when PD were dilated. In these experiments, we used an “empty” binary vector as a control.

When leaves expressing 35S-2xGFP//35S-RFP:NLS and 35S-KPILP were examined, 43% of the signal was distributed in clusters of 2, 3 or more cells, indicating that 2×GFP cell-to-cell movement was enhanced. Specifically, whereas only 5% of the signal was found in clusters of at least 3 cells in the control leaves, this value increased up to 21% in the presence of KPILP (**Table 1**). Student’s t-tests confirmed the statistical significance of the differences in the cell-to-cell movement of 2×GFP between the control and *KPILP*-expressing leaves.

**Table 1:** Quantification of 2×GFP intercellular movement^#^ in presence of 35S-KPILP or its mutant variants.

	1 cell	2 cells	≥3 cells
control	86 ± 5.0 (*)	9 ± 2.7 (*)	5 ± 2.5 (*)
KPILP	58 ± 1.5	22 ± 1.0	21 ± 1.2
KPILP (N60A)	76 ± 2.3 (*)	17 ± 1.3 (*)	7 ± 1.6 (*)
KPILP (N86A)	81 ± 2.9 (*)	15 ± 1.8 (*)	4 ± 1.3 (*)
KPILP (N136A)	80 ± 1.1 (*)	13 ± 0.8 (*)	6 ± 0.8 (*)

^#^The data shown in % of total number of clusters and represent four independent experiments with no less than five biological repeats and 200 cell clusters per sample analyzed. Means and standard error are indicated. *, p <0.05 (Student’s t-test) for statistical significance of the difference between KPILP and other variants.

### KPILP *N*-glycosylation is indispensable for its ability to activate intercellular transport

**Figure 6:**
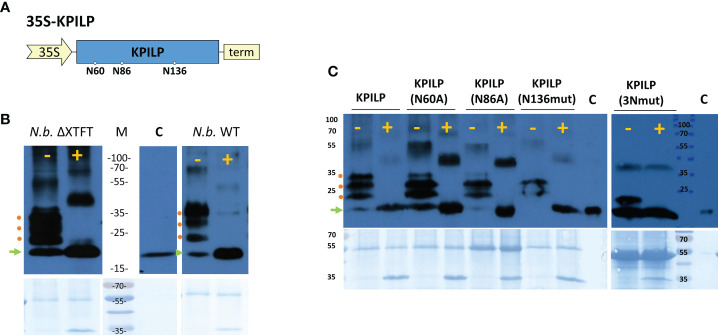
KPILP is a glycoprotein containing N-glycosylation sites sensitive to PNGase F treatment. **(A)** Schematic representation of genetic construct encoding KPILP. Predicted N-glycosylation sites are designated with circles. 35S, Cauliflower mosaic virus (CaMV) 35S promoter; term, 35S terminator of transcription. **(B)** Western blot analysis of KPILP-enriched preparation isolated from the leaves of transgenic ΔXTFT or wild-type (WT) *N. benthamiana* plants agroinfiltrated with 35S-KPILP. Samples treated with PNGase F (+) or without treatment (-) are probed with antibodies against KPILP. The lower panels show the protein loading control stained with Amido black. Protein molecular weight markers are indicated. C (control), recombinant 6×His-KPILP protein produced in *E*. *coli*. **(C)** KPILP-enriched preparation isolated from the leaves of wild-type *N. benthamiana* plants producing KPILP mutant variants with abolished one of the N-glycosylation sites or all of three of them (3Nmut). Samples treated with PNGase F (+) or without treatment (-) are probed with antibodies against KPILP raised in rabbit and then with anti-rabbit antibodies conjugated with horseradish peroxidase. The lower panels show the protein loading control stained with Amido black. Protein molecular weight markers are indicated. C (control), recombinant 6×His-KPILP protein produced in *E*. *coli*. Green arrowhead indicates the position of the non-glycosylated KPILP, red circles – KPILP with one or several N-linked glycans.

We previously suggested that KPILP undergoes glycosylation as it contains an N-terminal signal sequence that directs it to the endoplasmic reticulum/Golgi apparatus and putative N-glycosylation sites at Asn-60, Asn-86, and Asn-136 (**Figure 6A**) according to a prediction by the NetNGlyc service (http://www.cbs.dtu.dk/services/NetNGlyc/) (Sheshukova et al., 2017). Here, to confirm presence of Asn-linked glycans in KPILP, we used peptide-*N*-glycosidase F (PNGase F) (Plummer et al., 1984), which cleaves a bond between the innermost GlcNAc and asparagine residues in *N*-linked glycoproteins, except when the core GlcNAc is α1,3-fucosylated (Shen et al., 2017). This cleavage results in an increase of protein electrophoretic mobility. Therefore, to test the sensitivity to PNGase F, we used KPILP-enriched preparations of membrane proteins obtained from wild-type (WT) *N. benthamiana* plants and transgenic ΔXTFT *N. benthamiana* plants with knocked out β-1,2-xylosyltransferase (ΔXT) and α-1,3-fucosyltransferase (ΔFT) genes. The PNGase-treated samples were analyzed by Western blotting with antibodies against KPILP. KPILP-enriched protein preparations from *N. benthamiana* WT and ΔXTFT plants did not differ in sensitivity to PNGase F (**Figure 6B**), thus for further PNGase F treatments we used preparations from the WT *N. benthamiana* plants. Western blot demonstrates presence of at least 4 KPILP bands in the gel, which, after treatment with PNGase F, turn into a single major band with lower molecular weight that matches by the mobility to the protein band of the recombinant 6His-KPILP produced in *E. coli* (**Figure 6B**). This indicates that KPILP is a glycoprotein containing N-linked glycans sensitive to PNGase F.

At the next stage of the study, we created genetic constructs containing *KPILP* sequence with point mutations that abolished either potential KPILP N-glycosylation site. In the KPILP(N60A) and KPILP(N86A) mutants, the corresponding asparagine residue was substituted with alanine, while in the KPILP(N136mut) variant, we changed the amino acid context of Asn136 to prevent glycosylation. Moreover, we also created a triple mutant (3Nmut). Then, proteins isolated from *N. benthamiana* leaves agroinfiltrated with the described plasmids were treated with PNGase F and analyzed by Western blot with antibodies to KPILP (**Figure 6C**). Deleting the N-glycosylation sites N-60 and N-86 does not completely abolish KPILP glycosylation but changes the electrophoretic profile (**Figure 6C**). The simultaneous elimination of the three sites, including N-60, N-86 and N-136, also does not completely prevent KPILP glycosylation, which suggests possible glycosylation at other potential sites.

To determine how KPILP glycosylation affects its ability to participate in PD permeability regulation, we compared KPILP mutant variants in a test using 2×GFP reporter molecule. **Table 1** shows that introducing a mutation into any of the N-glycosylation site leads to a decrease in GFP-containing multicellular clusters to levels similar to control.

We concluded that the preservation of the native KPILP glycosylation profile and its secretion *via* the ER–Golgi pathway is necessary for its performance of PD gating functions.

## Discussion

Viral infection induces multiple changes in host plant cells. Among them are significant modifications of photosynthetic activity and chloroplast functioning (Ganusova et al., 2017; Bhattacharyya and Chakraborty, 2018; Souza et al., 2019; Navarro et al., 2021) that are caused directly by the viral proteins (Reinero and Beachy, 1989; Qiao et al., 2009) or indirectly as a consequence of the re-formatting and functional changes in the cell, i.e. side-effects of viral reproduction (Bhat et al., 2013; Li et al., 2016; Budziszewska and Obrępalska-Stęplowska, 2018). Also during viral infection, the intercellular transport of macromolecules is upregulated by the viral and cellular factors.

Here, we studied the role of stress-induced *KPILP* gene in plant-virus interaction. We designed a model system that allows to monitor the effects of *KPILP* up- and downregulation on the background of PVX infection: *KPILP* expression increases in response to viral infection *per se* and to obtain the decreased *KPILP* mRNA levels during PVX infection we used VIGS approach exploiting PVX-based vector both to create the conditions of viral infection and to induce *KPILP* silencing. We have demonstrated that *KPILP* is involved in CRS regulatory hub during viral infection as it participates in the suppression of nuclear encoded chloroplast genes *LHCB1* and *LHCB2*, *RBCS1A* and *HEMA1*. Similar effect on the expression of the studied PhANGs was revealed in *N. benthamiana* plants with reduced *ISE2* level (Ganusova et al., 2020) and in the well characterized Arabidopsis mutant *ISE2* embryos that are characterized with impaired photosynthesis and are lethal due to the suppression of genes involved in the LHC formation as well as in chlorophyll synthesis and carbohydrates production (Burch-Smith et al., 2011a). However, in our study the level of *ISE2* mRNA remained the same either in pPVX- or pPVX(frKPILP)-infected plants compared with intact plants (data not shown). Together these results indicate that *KPILP* is involved in the suppression of photosynthesis and carbohydrate metabolism during viral infection in an *ISE2*-independent pathway.

Taking into account that *KPILP* is known to be upregulated in response to prolonged darkness (Sheshukova et al., 2017; Sheshukova et al., 2018) we could suggest that its activation during viral infection engages the same mechanisms that underlie the darkness-induced activation and both pathways are connected with cellular photosynthetic activity and PhANG expression.

During PVX infection the level of produced and accumulated photoassimilates is significantly decreased. Our results indicate that *KPILP* participates in glucose partitioning and carbohydrate metabolism in PVX-infected leaves as in *KPILP*-silenced plants the level of glucose in sink leaves is 2-fold higher than in sink leaves with upregulated *KPILP*, moreover, in source leaves of *KPILP*-silenced plants glucose content is 3-fold higher compared to source leaves with increased *KPILP* expression. These results allow us to conclude that *KPILP* negatively affects photoassimilates accumulation during viral infection suppressing chloroplast nuclear-encoded genes that determine the physiological status of chloroplasts and triggering CRS. Viral reproduction and activation of antiviral defense, such as viral RNA silencing, is a very energy-consuming process (Nagy and Lin, 2020). Metabolic changes occurring in infected tissues lead to the depletion of photoassimilates necessary for the synthesis of new proteins and energy for biosynthetic processes (Zhao et al., 2016). Chloroplasts are not only the source of photoassimilates but also they generate various forms of ROS, which are strategic activators of defense pathways in cells (Kuźniak and Kopczewski, 2020). During viral infection we observe genetic and metabolic changes in chloroplasts associated with the suppression of carbohydrates synthesis. These effects could be a consequence of the redistribution of energy resources from normal metabolism to the activation of plant defense reactions followed by virus-induced suppression of these defense reactions and utilization of the cellular resources for the viral reproduction. Although PVX genomic RNA levels did not differ between analyzed experimental groups (**Figure S2A**), PVX CP accumulated less efficiently in plants with *KPILP* silencing (**Figure S2B**). However, the question about *KPILP* negative effect on antiviral defense reactions remains open and requires further study.

In PVX-infected plants with *KPILP* knockdown we observed a decreased amount of mature PsbQ protein, a component of photosystem II oxygen-evolving complex. We suggested that during PVX infection one or several viral proteins interact with PsbQ precursor exploiting its chloroplast transit peptide to enter chloroplast and photosynthetic apparatus while KPILP is essential for this interaction. A similar interaction with chloroplast proteins has been described for PVX CP that interacts with plastocyanin transit peptide and thus enters the chloroplast (Qiao et al., 2009). It is possible that *KPILP* downregulation leads to less effective penetration of viral protein(s) into the chloroplast which results to milder suppression of the CRS marker genes and carbohydrate metabolism.

Another *KPILP* function is associated with PD control. We demonstrated that *KPILP* stimulates intercellular transport of reporter 2xGFP molecules being a positive PD regulator. Moreover, natural KPILP glycosylation is indispensable for manifestation of this function. And during PVX infection *KPILP* increased expression leads to the decrease in PD callose deposition. We have demonstrated that increased *KPILP* expression stimulates intercellular transport and correlates with *XTH5* expression. *XTHs* were shown to be upregulated in *ISE2* mutants (Burch-Smith et al., 2011a) or in plants with *ISE2* knockdown (Ganusova et al., 2020) both characterized with the increased intercellular traffic. The *XTH* genes comprise a large group of genes the expression of which is organ-specific and depends on the stage of development (Yokoyama and Nishitani, 2001; Ishida and Yokoyama, 2022). The transglycosylase activity of XTH towards xyloglucans has been long believed to contribute to the loosening of the cell wall during cell growth (Cosgrove, 2016). However, the recent data indicate that XTHs participate in adaptation to abiotic stresses, plant-virus interactions and cell wall structures remodeling (Shimizu et al., 2007; Otulak-Kozieł et al., 2018; Hrmova et al., 2022; Ishida and Yokoyama, 2022). Noteworthy, the expression of *XTHs* genes is regulated by retrograde signals in a light-dependent manner. As was demonstrated for *A. thaliana*, ABI4 transcription factor promotes *XTH5* expression, while HY5 inhibits it (Xu et al., 2016). Both these transcription factors participate in photomorphogenesis. Moreover, ABI4 is a key player in retrograde signaling pathways: it binds the promoter of a retrograde-regulated genes and downregulates PhANG in response to signals from aberrantly functioning plastids (Koussevitzky et al., 2007). HY5 regulates hypocotyl cell elongation and photomorphogenesis (Jing et al., 2013). During seedling de-etiolation ABI4 and HY5 have the antagonistic effects on photomorfogenesis (Xu et al., 2016). As genes for both transcription factors are found in *Nicotiana* sp., the homologues of ABI4 (Alazem et al., 2014) and HY5 (Oh et al., 2018) could probably function as regulators of *KPILP* expression in a light-dependent manner and in response to virus. In intact leaves both *KPILP* and *XTH5* expression is low but upregulated during viral infection. Thus, we could speculate that KPILP being a secretory protein affects XTH in the cell wall inducing increase of PD permeability *via* putative interaction of XTHs with the cell wall components, that are involved in the regulation of callose depositions under stress conditions. This could be either enzymes directly modifying callose, for example, callose synthases or beta-glucanases, or some factors regulation callose depositions indirectly (Zavaliev et al., 2011; Wu et al., 2018).

Viral infection leads to the suppression of the genes encoding essential components of leaf photosynthetic apparatus. On the other hand, chloroplasts are involved in antiviral immune defense (Nomura et al., 2012; Serrano et al., 2016; Medina-Puche et al., 2020). Obviously, the pattern of gene expression in photosynthetic and non-photosynthetic tissues is fundamentally different. Genes active in roots that are not exposed to light are not active in leaves and photosynthesis-related genes are suppressed there. One of the successful strategies for viral infection could be the strategy of “switching off” photosynthetic genes through the induction of expression of root regulatory genes in order to suppress antiviral immunity triggered by retrograde signals from chloroplasts. In addition to affecting the plant immune system, the virus can also modulate the expression of genes that regulate cell-to-cell and long distance transport in the same way, thus facilitating viral reproduction and spread of the infection. Based on this hypothesis, our results presented in this article are integrated into a model that describes organelle-nucleus-PD signaling (ONPS) through the activation of *KPILP* expression under conditions of viral infection. Under normal conditions and in the absence of stressful impact, the expression of *KPILP* is downregulated, chloroplasts transmit biogenic signals to the nucleus, indicating a normal physiological status, carbon metabolism and PD-transport of sugars and other factors occur normally.

In virus-infected leaves *KPILP* expression is activated and that launches a regulatory pathway in which the expression of some PhANGs downregulated and the expression of genes participating in PD-regulation is triggered. PhANG suppression leads to the decrease of ROS and hormones production by chloroplasts and weakening of the antiviral defense. PDANG upregulation results in an activation of cell-to-cell and, consequently, long-distance transport, creating favorable conditions for reproduction and spread of viral infection.

## Data availability statement

The original contributions presented in the study are included in the article/**Supplementary Material**. Further inquiries can be directed to the corresponding author.

## Author contributions

NE, ES, TK designed and performed most of the experiments, NE, TK analyzed the data, prepared figures; KK obtained genetic constructs; EA assisted in confocal microscopy imaging; VT quantified glucose content; MS performed mass-spectroscopy and protein analysis. NE and TK developed the general concept, supervised the study and wrote the manuscript. All authors contributed to the article and approved the submitted version.

## Funding

The study was supported by the Russian Science Foundation (project No. 19-74-20031).

## Acknowledgments

The authors thank Lomonosov Moscow State University Development Program PNR5.13 and IBCH core facility (CKP IBCH of the Shemyakin-Ovchinnikov Institute of Bioorganic Chemistry, Russian Academy of Sciences) for providing access to the scientific instruments. The authors are particularly grateful to Dr. Yuri Dorokhov for the inspiring ideas and the impact in the research conceptualization.

## Conflict of interest

The authors declare that the research was conducted in the absence of any commercial or financial relationships that could be construed as a potential conflict of interest.

## Publisher’s note

All claims expressed in this article are solely those of the authors and do not necessarily represent those of their affiliated organizations, or those of the publisher, the editors and the reviewers. Any product that may be evaluated in this article, or claim that may be made by its manufacturer, is not guaranteed or endorsed by the publisher.

## References

[B1] Alazem M. Lin K.-Y. Lin N.-S. (2014). The abscisic acid pathway has multifaceted effects on the accumulation of bamboo mosaic virus. Mol. Plant-Microbe Interact. MPMI 27, 177–189. doi: 10.1094/MPMI-08-13-0216-R 24224533

[B2] Azim M. F. Burch-Smith T. M. (2020). Organelles-nucleus-plasmodesmata signaling (ONPS): an update on its roles in plant physiology, metabolism and stress responses. Curr. Opin. Plant Biol. 58, 48–59. doi: 10.1016/j.pbi.2020.09.005 33197746

[B3] Baulcombe D. C. Chapman S. Santa Cruz S. (1995). Jellyfish green fluorescent protein as a reporter for virus infections. Plant J. Cell Mol. Biol. 7, 1045–1053. doi: 10.1046/j.1365-313x.1995.07061045.x 7599646

[B4] Bhat S. Folimonova S. Y. Cole A. B. Ballard K. D. Lei Z. Watson B. S. . (2013). Influence of host chloroplast proteins on tobacco mosaic virus accumulation and intercellular movement. Plant Physiol. 161, 134–147. doi: 10.1104/pp.112.207860 23096159 PMC3532247

[B5] Bhattacharyya D. Chakraborty S. (2018). Chloroplast: the Trojan horse in plant-virus interaction. Mol. Plant Pathol. 19, 504–518. doi: 10.1111/mpp.12533 28056496 PMC6638057

[B6] Bilgin D. D. Zavala J. A. Zhu J. Clough S. J. Ort D. R. De Lucia E. H. (2010). Biotic stress globally downregulates photosynthesis genes. Plant Cell Environ. 33, 1597–1613. doi: 10.1111/j.1365-3040.2010.02167.x 20444224

[B7] Bobik K. McCray T. N. Ernest B. Fernandez J. C. Howell K. A. Lane T. . (2017). The chloroplast RNA helicase *ISE2* is required for multiple chloroplast RNA processing steps in arabidopsis thaliana. Plant J. Cell Mol. Biol. 91, 114–131. doi: 10.1111/tpj.13550 28346704

[B8] Brunkard J. O. Burch-Smith T. M. (2018). Ties that bind: the integration of plastid signalling pathways in plant cell metabolism. Essays Biochem. 62, 95–107. doi: 10.1042/EBC20170011 29563221 PMC6082656

[B9] Brunkard J. O. Zambryski P. C. (2017). Plasmodesmata enable multicellularity: new insights into their evolution, biogenesis, and functions in development and immunity. Curr. Opin. Plant Biol. 35, 76–83. doi: 10.1016/j.pbi.2016.11.007 27889635

[B10] Budziszewska M. Obrępalska-Stęplowska A. (2018). The role of the chloroplast in the replication of positive-sense single-stranded plant RNA viruses. Front. Plant Sci. 9. doi: 10.3389/fpls.2018.01776 PMC6278097 30542365

[B11] Burch-Smith T. M. Brunkard J. O. Choi Y. G. Zambryski P. C. (2011a). Organelle–nucleus cross-talk regulates plant intercellular communication via plasmodesmata. Proc. Natl. Acad. Sci. 108, E1451–E1460. doi: 10.1073/pnas.1117226108 22106293 PMC3251100

[B12] Burch-Smith T. M. Stonebloom S. Xu M. Zambryski P. C. (2011b). Plasmodesmata during development: re-examination of the importance of primary, secondary, and branched plasmodesmata structure versus function. Protoplasma 248, 61–74. doi: 10.1007/s00709-010-0252-3 21174132 PMC3025111

[B13] Burch-Smith T. M. Zambryski P. C. (2010). Loss of INCREASED SIZE EXCLUSION LIMIT (*ISE*)1 or *ISE2* increases the formation of secondary plasmodesmata. Curr. Biol. 20, 989–993. doi: 10.1016/j.cub.2010.03.064 20434343 PMC2902234

[B14] Burch-Smith T. M. Zambryski P. C. (2012). Plasmodesmata paradigm shift: regulation from without versus within. Annu. Rev. Plant Biol. 63, 239–260. doi: 10.1146/annurev-arplant-042811-105453 22136566

[B15] Burton R. A. Gibeaut D. M. Bacic A. Findlay K. Roberts K. Hamilton A. . (2000). Virus-induced silencing of a plant cellulose synthase gene. Plant Cell 12, 691–706. doi: 10.1105/tpc.12.5.691 10810144 PMC139921

[B16] Caplan J. L. Mamillapalli P. Burch-Smith T. M. Czymmek K. Dinesh-Kumar S. P. (2008). Chloroplastic protein NRIP1 mediates innate immune receptor recognition of a viral effector. Cell 132, 449–462. doi: 10.1016/j.cell.2007.12.031 18267075 PMC2267721

[B17] Cosgrove D. J. (2016). Catalysts of plant cell wall loosening. F1000Research 5, F1000 Faculty Rev–119. doi: 10.12688/f1000research.7180.1 PMC4755413 26918182

[B18] Crawford T. Lehotai N. Strand Å. (2018). The role of retrograde signals during plant stress responses. J. Exp. Bot. 69, 2783–2795. doi: 10.1093/jxb/erx481 29281071

[B19] Crawford K. M. Zambryski P. C. (2000). Subcellular localization determines the availability of non-targeted proteins to plasmodesmatal transport. Curr. Biol. CB 10, 1032–1040. doi: 10.1016/s0960-9822(00)00657-6 10996070

[B20] Dorokhov Y. L. Ershova N. M. Sheshukova E. V. Komarova T. V. (2019). Plasmodesmata conductivity regulation: A mechanistic model. Plants Basel Switz. 8, 595. doi: 10.3390/plants8120595 PMC6963776 31842374

[B21] Dorokhov Y. L. Komarova T. V. Petrunia I. V. Frolova O. Y. Pozdyshev D. V. Gleba Y. Y. (2012). Airborne signals from a wounded leaf facilitate viral spreading and induce antibacterial resistance in neighboring plants. PloS Pathog. 8, e1002640. doi: 10.1371/journal.ppat.1002640 22496658 PMC3320592

[B22] Faulkner C. (2018). Plasmodesmata and the symplast. Curr. Biol. CB 28, R1374–R1378. doi: 10.1016/j.cub.2018.11.004 30562524

[B23] Fitter D. W. Martin D. J. Copley M. J. Scotland R. W. Langdale J. A. (2002). GLK gene pairs regulate chloroplast development in diverse plant species. Plant J. Cell Mol. Biol. 31, 713–727. doi: 10.1046/j.1365-313x.2002.01390.x 12220263

[B24] Ganusova E. E. Reagan B. C. Fernandez J. C. Azim M. F. Sankoh A. F. Freeman K. M. . (2020). Chloroplast-to-nucleus retrograde signalling controls intercellular trafficking *via* plasmodesmata formation. Philos. Trans. R. Soc B Biol. Sci. 375, 20190408. doi: 10.1098/rstb.2019.0408 PMC7209952 32362251

[B25] Ganusova E. Rice J. H. Carlew T. S. Patel A. Perrodin-Njoku E. Hewezi T. . (2017). Altered expression of a chloroplast protein affects the outcome of virus and nematode infection. Mol. Plant-Microbe Interact. MPMI 30, 478–488. doi: 10.1094/MPMI-02-17-0031-R 28323529

[B26] Hrmova M. Stratilová B. Stratilová E. (2022). Broad specific Xyloglucan:Xyloglucosyl transferases are formidable players in the re-modelling of plant cell wall structures. Int. J. Mol. Sci. 23, 1656. doi: 10.3390/ijms23031656 35163576 PMC8836008

[B27] Inaba T. Ito-Inaba Y. (2010). Versatile roles of plastids in plant growth and development. Plant Cell Physiol. 51, 1847–1853. doi: 10.1093/pcp/pcq147 20889507

[B28] Ishida K. Yokoyama R. (2022). Reconsidering the function of the xyloglucan endotransglucosylase/hydrolase family. J. Plant Res. 135, 145–156. doi: 10.1007/s10265-021-01361-w 35000024

[B29] Jing Y. Zhang D. Wang X. Tang W. Wang W. Huai J. . (2013). Arabidopsis chromatin remodeling factor PICKLE interacts with transcription factor HY5 to regulate hypocotyl cell elongation. Plant Cell 25, 242–256. doi: 10.1105/tpc.112.105742 23314848 PMC3584539

[B30] Kakizaki T. Matsumura H. Nakayama K. Che F.-S. Terauchi R. Inaba T. (2009). Coordination of plastid protein import and nuclear gene expression by plastid-to-nucleus retrograde signaling. Plant Physiol. 151, 1339–1353. doi: 10.1104/pp.109.145987 19726569 PMC2773054

[B31] Kim I. Hempel F. D. Sha K. Pfluger J. Zambryski P. C. (2002). Identification of a developmental transition in plasmodesmatal function during embryogenesis in arabidopsis thaliana. Dev. Camb. Engl. 129, 1261–1272. doi: 10.1242/dev.129.5.1261 11874921

[B32] Kobayashi K. Otegui M. S. Krishnakumar S. Mindrinos M. Zambryski P. (2007). INCREASED SIZE EXCLUSION LIMIT2 encodes a putative DEVH box RNA helicase involved in plasmodesmata function during arabidopsis embryogenesis. Plant Cell 19, 1885–1897. doi: 10.1105/tpc.106.045666 17601829 PMC1955720

[B33] Komarova T. V. Skulachev M. V. Zvereva A. S. Schwartz A. M. Dorokhov Y. Atabekov J. G. (2006). New viral vector for efficient production of target proteins in plants. Biochem. Mosc. 71, 846–850. doi: 10.1134/S0006297906080049 16978146

[B34] Koussevitzky S. Nott A. Mockler T. C. Hong F. Sachetto-Martins G. Surpin M. . (2007). Signals from chloroplasts converge to regulate nuclear gene expression. Science 316, 715–719. doi: 10.1126/science.1140516 17395793

[B35] Kuźniak E. Kopczewski T. (2020). The chloroplast reactive oxygen species-redox system in plant immunity and disease. Front. Plant Sci. 11, 572686. doi: 10.3389/fpls.2020.572686 33281842 PMC7688986

[B36] Laliberté J.-F. Sanfaçon H. (2010). Cellular remodeling during plant virus infection. Annu. Rev. Phytopathol. 48, 69–91. doi: 10.1146/annurev-phyto-073009-114239 20337516

[B37] Li Y. Cui H. Cui X. Wang A. (2016). The altered photosynthetic machinery during compatible virus infection. Curr. Opin. Virol. 17, 19–24. doi: 10.1016/j.coviro.2015.11.002 26651024

[B38] Lucas W. J. Lee J.-Y. (2004). Plasmodesmata as a supracellular control network in plants. Nat. Rev. Mol. Cell Biol. 5, 712–726. doi: 10.1038/nrm1470 15340379

[B39] Medina-Puche L. Tan H. Dogra V. Wu M. Rosas-Diaz T. Wang L. . (2020). A defense pathway linking plasma membrane and chloroplasts and Co-opted by pathogens. Cell 182, 1109–1124.e25. doi: 10.1016/j.cell.2020.07.020 32841601

[B40] Nagy P. D. Lin W. (2020). Taking over cellular energy-metabolism for TBSV replication: The high ATP requirement of an RNA virus within the viral replication organelle. Viruses 12, 56. doi: 10.3390/v12010056 31947719 PMC7019945

[B41] Navarro J. A. Saiz-Bonilla M. Sanchez-Navarro J. A. Pallas V. (2021). The mitochondrial and chloroplast dual targeting of a multifunctional plant viral protein modulates chloroplast-to-nucleus communication, RNA silencing suppressor activity, encapsidation, pathogenesis and tissue tropism. Plant J. 108, 197–218. doi: 10.1111/tpj.15435 34309112

[B42] Nomura H. Komori T. Uemura S. Kanda Y. Shimotani K. Nakai K. . (2012). Chloroplast-mediated activation of plant immune signalling in arabidopsis. Nat. Commun. 3, 926. doi: 10.1038/ncomms1926 22735454

[B43] Oh Y. Fragoso V. Guzzonato F. Kim S.-G. Park C.-M. Baldwin I. T. (2018). Root-expressed phytochromes B1 and B2, but not PhyA and Cry2, regulate shoot growth in nature. Plant Cell Environ. 41, 2577–2588. doi: 10.1111/pce.13341 29766532

[B44] Otulak-Kozieł K. Kozieł E. Bujarski J. J. (2018). Spatiotemporal changes in xylan-1/Xyloglucan and xyloglucan xyloglucosyl transferase (XTH-Xet5) as a step-in of ultrastructural cell wall remodelling in potato–potato virus y (PVYNTN) hypersensitive and susceptible reaction. Int. J. Mol. Sci. 19, 2287. doi: 10.3390/ijms19082287 30081556 PMC6121353

[B45] Patrick J. W. Botha F. C. Birch R. G. (2013). Metabolic engineering of sugars and simple sugar derivatives in plants. Plant Biotechnol. J. 11, 142–156. doi: 10.1111/pbi.12002 23043616

[B46] Pfaffl M. W. (2001). A new mathematical model for relative quantification in real-time RT–PCR. Nucleic Acids Res. 29, e45. doi: 10.1093/nar/29.9.e45 11328886 PMC55695

[B47] Pfannschmidt T. Terry M. J. Van Aken O. Quiros P. M. (2020). Retrograde signals from endosymbiotic organelles: a common control principle in eukaryotic cells. Philos. Trans. R. Soc B Biol. Sci. 375, 20190396. doi: 10.1098/rstb.2019.0396 PMC7209961 32362267

[B48] Plummer T. H. Elder J. H. Alexander S. Phelan A. W. Tarentino A. L. (1984). Demonstration of peptide:N-glycosidase F activity in endo-beta-N-acetylglucosaminidase F preparations. J. Biol. Chem. 259, 10700–10704. doi: 10.1016/S0021-9258(18)90568-5 6206060

[B49] Qiao Y. Li H. F. Wong S. M. Fan Z. F. (2009). Plastocyanin transit peptide interacts with *Potato virus x* coat protein, while silencing of plastocyanin reduces coat protein accumulation in chloroplasts and symptom severity in host plants. Mol. Plant-Microbe Interact. 22, 1523–1534. doi: 10.1094/MPMI-22-12-1523 19888818

[B50] Reinero A. Beachy R. N. (1989). Reduced photosystem II activity and accumulation of viral coat protein in chloroplasts of leaves infected with tobacco mosaic virus. Plant Physiol. 89, 111–116. doi: 10.1104/pp.89.1.111 16666500 PMC1055805

[B51] Sager R. Lee J.-Y. (2014). Plasmodesmata in integrated cell signalling: insights from development and environmental signals and stresses. J. Exp. Bot. 65, 6337–6358. doi: 10.1093/jxb/eru365 25262225 PMC4303807

[B52] Sager R. E. Lee J.-Y. (2018). Plasmodesmata at a glance. J. Cell Sci. 131, jcs209346. doi: 10.1242/jcs.209346 29880547

[B53] Schmied J. Hedtke B. Grimm B. (2011). Overexpression of HEMA1 encoding glutamyl-tRNA reductase. J. Plant Physiol. 168, 1372–1379. doi: 10.1016/j.jplph.2010.12.010 21272955

[B54] Serrano I. Audran C. Rivas S. (2016). Chloroplasts at work during plant innate immunity. J. Exp. Bot. 67, 3845–3854. doi: 10.1093/jxb/erw088 26994477

[B55] Shen W. Xiao Z. Shen J. Gao C. (2017). Analysis of golgi-mediated protein traffic in plant cells. Methods Mol. Biol. Clifton NJ 1662, 75–86. doi: 10.1007/978-1-4939-7262-3_6 28861818

[B56] Sheshukova E. V. Komarova T. V. Ershova N. M. Bronstein A. M. Dorokhov Y. L. (2018). The expression of matryoshka gene encoding a homologue of kunitz peptidase inhibitor is regulated both at the level of transcription and translation. Biochem. Mosc. 83, 1255–1262. doi: 10.1134/S0006297918100103 30472962

[B57] Sheshukova E. V. Komarova T. V. Ershova N. M. Shindyapina A. V. Dorokhov Y. L. (2017). An alternative nested reading frame may participate in the stress-dependent expression of a plant gene. Front. Plant Sci. 8. doi: 10.3389/fpls.2017.02137 PMC5742262 29312392

[B58] Shimizu T. Satoh K. Kikuchi S. Omura T. (2007). The repression of cell wall- and plastid-related genes and the induction of defense-related genes in rice plants infected with rice dwarf virus. Mol. Plant-Microbe Interact. MPMI 20, 247–254. doi: 10.1094/MPMI-20-3-0247 17378427

[B59] Shimizu T. Yoshii A. Sakurai K. Hamada K. Yamaji Y. Suzuki M. . (2009). Identification of a novel tobacco DnaJ-like protein that interacts with the movement protein of tobacco mosaic virus. Arch. Virol. 154, 959–967. doi: 10.1007/s00705-009-0397-6 19458900

[B60] Souza P. F. N. Garcia-Ruiz H. Carvalho F. E. L. (2019). What proteomics can reveal about plant–virus interactions? photosynthesis-related proteins on the spotlight. Theor. Exp. Plant Physiol. 31, 227–248. doi: 10.1007/s40626-019-00142-0 31355128 PMC6660014

[B61] Strasser R. Stadlmann J. Schähs M. Stiegler G. Quendler H. Mach L. . (2008). Generation of glyco-engineered nicotiana benthamiana for the production of monoclonal antibodies with a homogeneous human-like n-glycan structure. Plant Biotechnol. J. 6, 392–402. doi: 10.1111/j.1467-7652.2008.00330.x 18346095

[B62] Sun Y. Huang D. Chen X. (2019). Dynamic regulation of plasmodesmatal permeability and its application to horticultural research. Hortic. Res. 6, 47. doi: 10.1038/s41438-019-0129-3 30962940 PMC6441653

[B63] Susek R. E. Ausubel F. M. Chory J. (1993). Signal transduction mutants of arabidopsis uncouple nuclear CAB and RBCS gene expression from chloroplast development. Cell 74, 787–799. doi: 10.1016/0092-8674(93)90459-4 7690685

[B64] Tabler M. Tsagris M. (2004). Viroids: petite RNA pathogens with distinguished talents. Trends Plant Sci. 9, 339–348. doi: 10.1016/j.tplants.2004.05.007 15231279

[B65] Tilsner J. Nicolas W. Rosado A. Bayer E. M. (2016). Staying tight: Plasmodesmal membrane contact sites and the control of cell-to-Cell connectivity in plants. Annu. Rev. Plant Biol. 67, 337–364. doi: 10.1146/annurev-arplant-043015-111840 26905652

[B66] Timmermans M. C. P. Maliga P. Vieira J. Messing J. (1990). The pFF plasmids: cassettes utilising CaMV sequences for expression of foreign genes in plants. J. Biotechnol. 14, 333–344. doi: 10.1016/0168-1656(90)90117-T 1369289

[B67] Wu S.-W. Kumar R. Iswanto A. B. B. Kim J.-Y. (2018). Callose balancing at plasmodesmata. J. Exp. Bot. 69, 5325–5339. doi: 10.1093/jxb/ery317 30165704

[B68] Xu X. Chi W. Sun X. Feng P. Guo H. Li J. . (2016). Convergence of light and chloroplast signals for de-etiolation through ABI4-HY5 and COP1. Nat. Plants 2, 16066. doi: 10.1038/nplants.2016.66 27255835

[B69] Yokoyama R. Nishitani K. (2001). A comprehensive expression analysis of all members of a gene family encoding cell-wall enzymes allowed us to predict cis-regulatory regions involved in cell-wall construction in specific organs of arabidopsis. Plant Cell Physiol. 42, 1025–1033. doi: 10.1093/pcp/pce154 11673616

[B70] Zavaliev R. Epel B. L. (2015). Imaging callose at plasmodesmata using aniline blue: quantitative confocal microscopy. Methods Mol. Biol. Clifton NJ 1217, 105–119. doi: 10.1007/978-1-4939-1523-1_7 25287199

[B71] Zavaliev R. Ueki S. Epel B. L. Citovsky V. (2011). Biology of callose (β-1,3-glucan) turnover at plasmodesmata. Protoplasma 248, 117–130. doi: 10.1007/s00709-010-0247-0 21116665 PMC9473521

[B72] Zhao J. Zhang X. Hong Y. Liu Y. (2016). Chloroplast in plant-virus interaction. Front. Microbiol. 7. doi: 10.3389/fmicb.2016.01565 PMC5047884 27757106

