# Supplementary figures and images for: Nicotiana benthamiana Kunitz peptidase inhibitor-like protein involved in chloroplast-to-nucleus regulatory pathway in plant-virus interaction
Source: Front Plant Sci. 2022 Nov 10;13:1041867. doi: 10.3389/fpls.2022.1041867 (PMC9685412; doi:10.3389/fpls.2022.1041867)

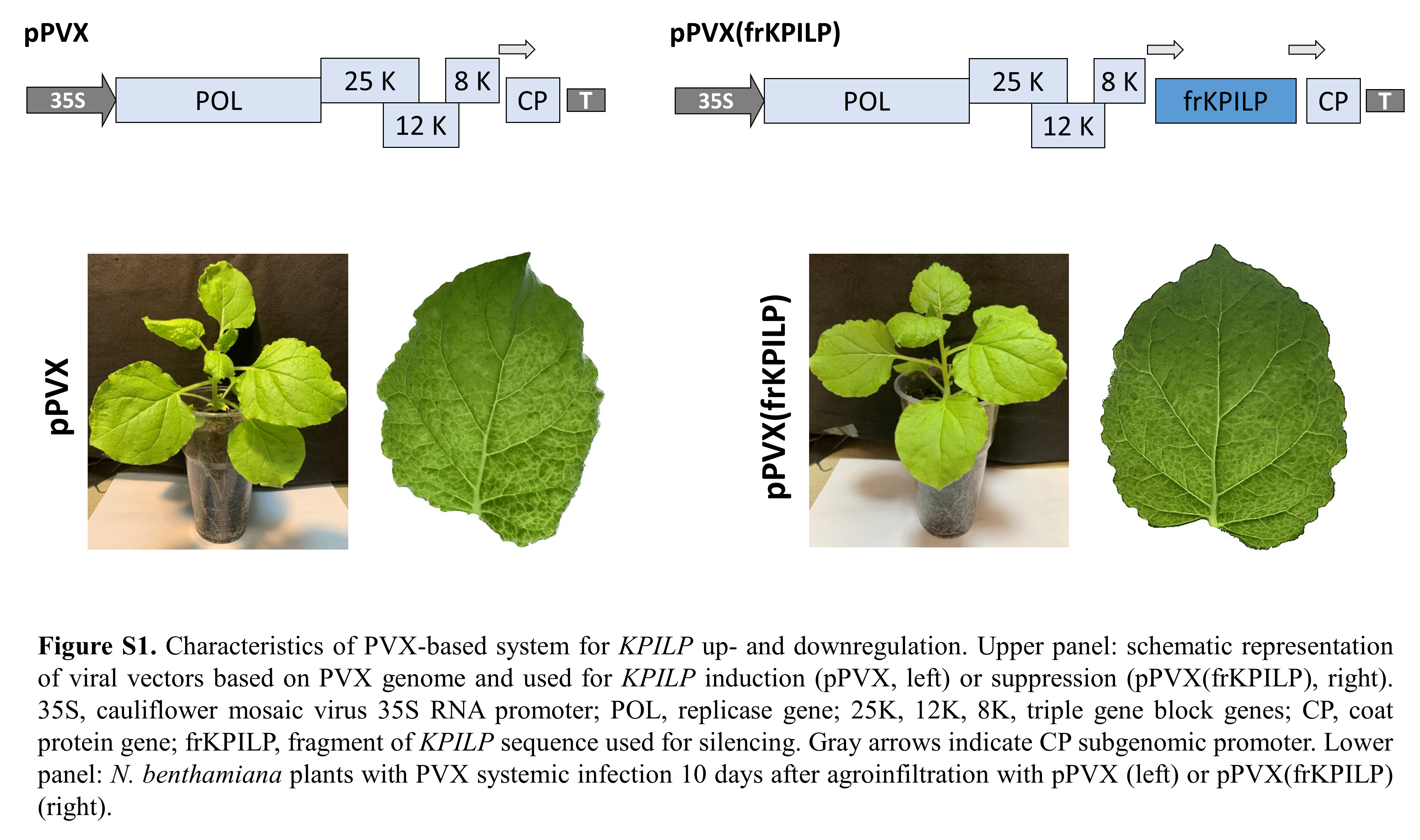

Supplement: Supplementary file 1 [file Image_1.tif]

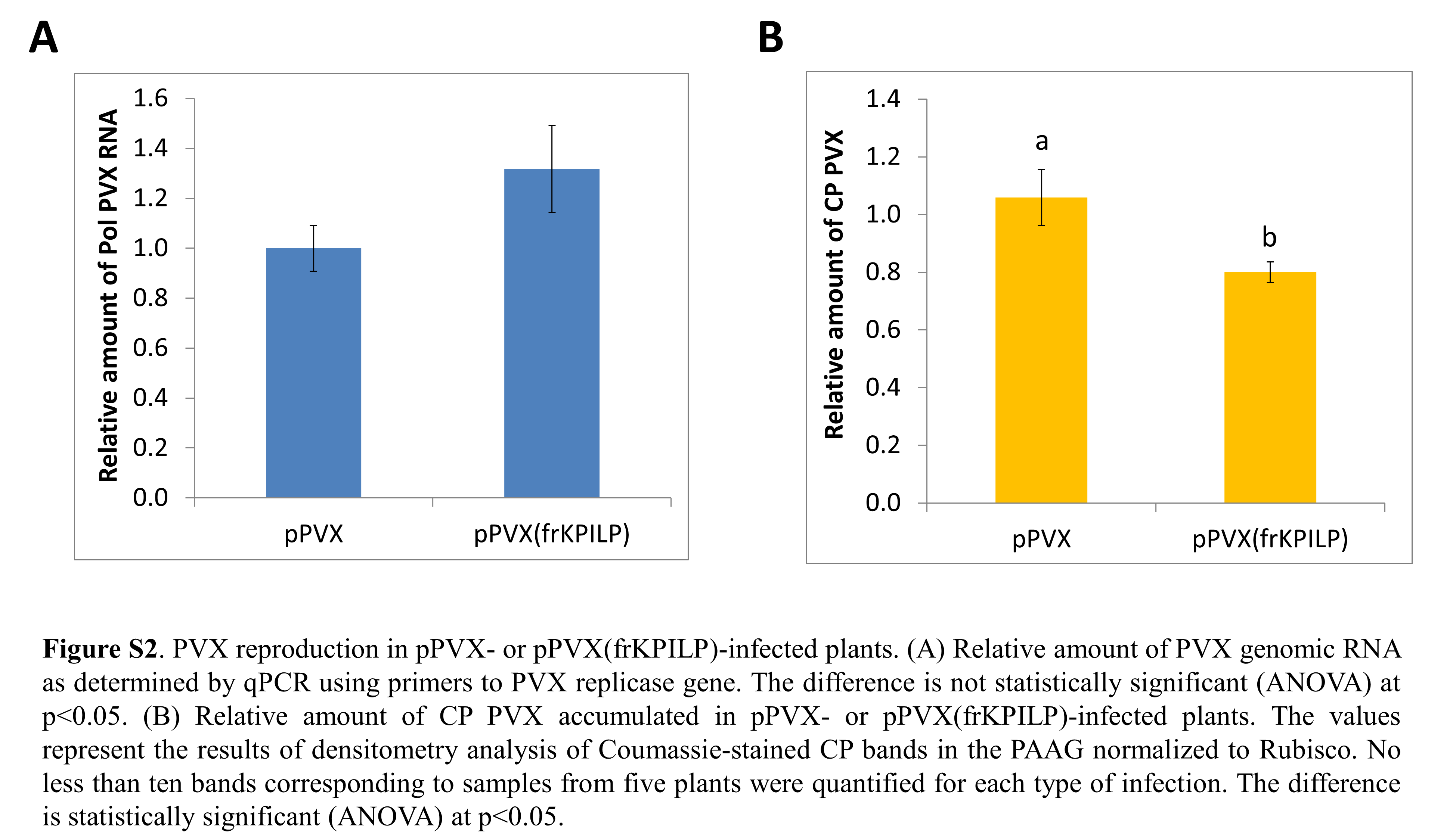

Supplement: Supplementary file 2 [file Image_2.tif]

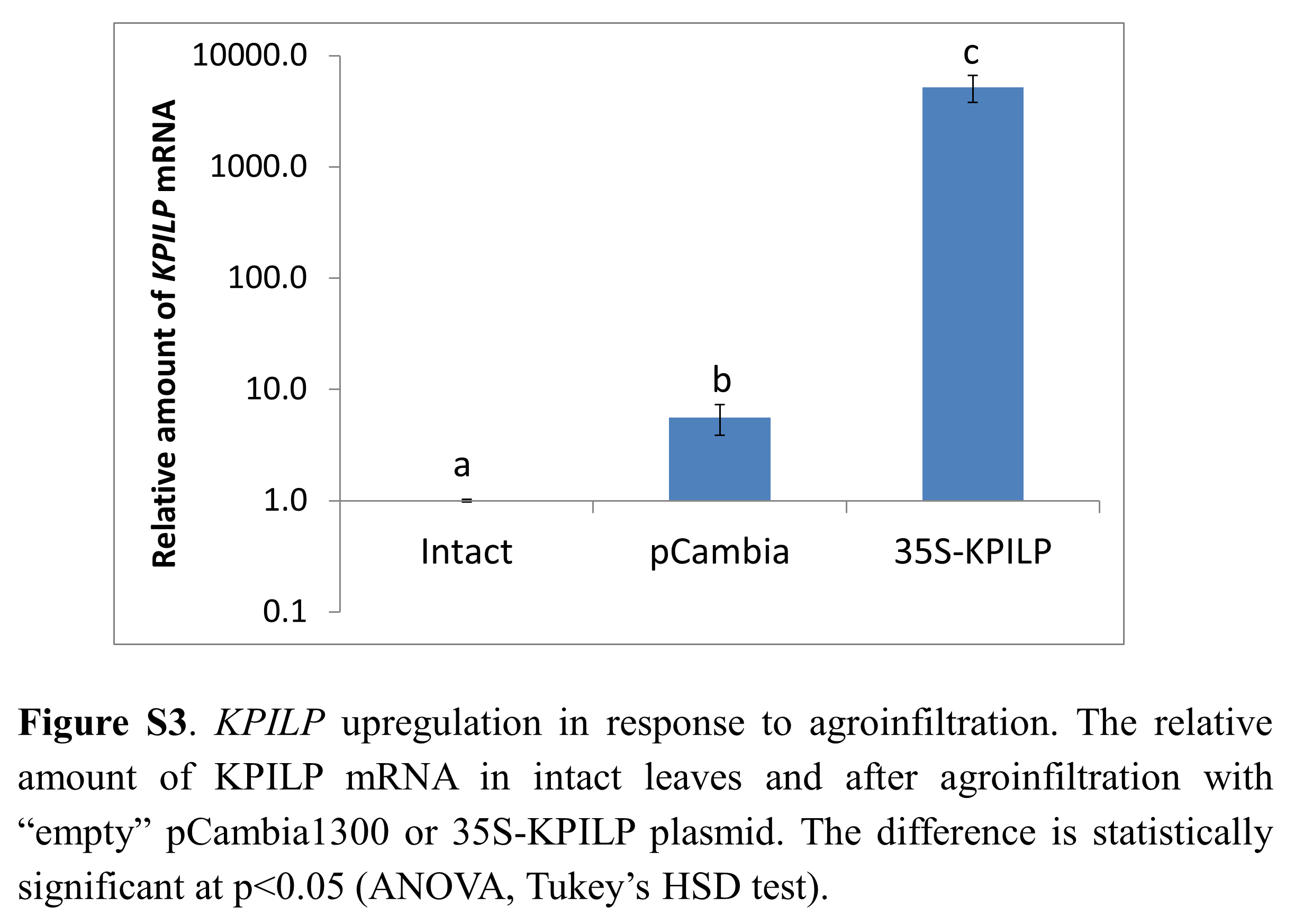

Supplement: Supplementary file 3 [file Image_3.tif]

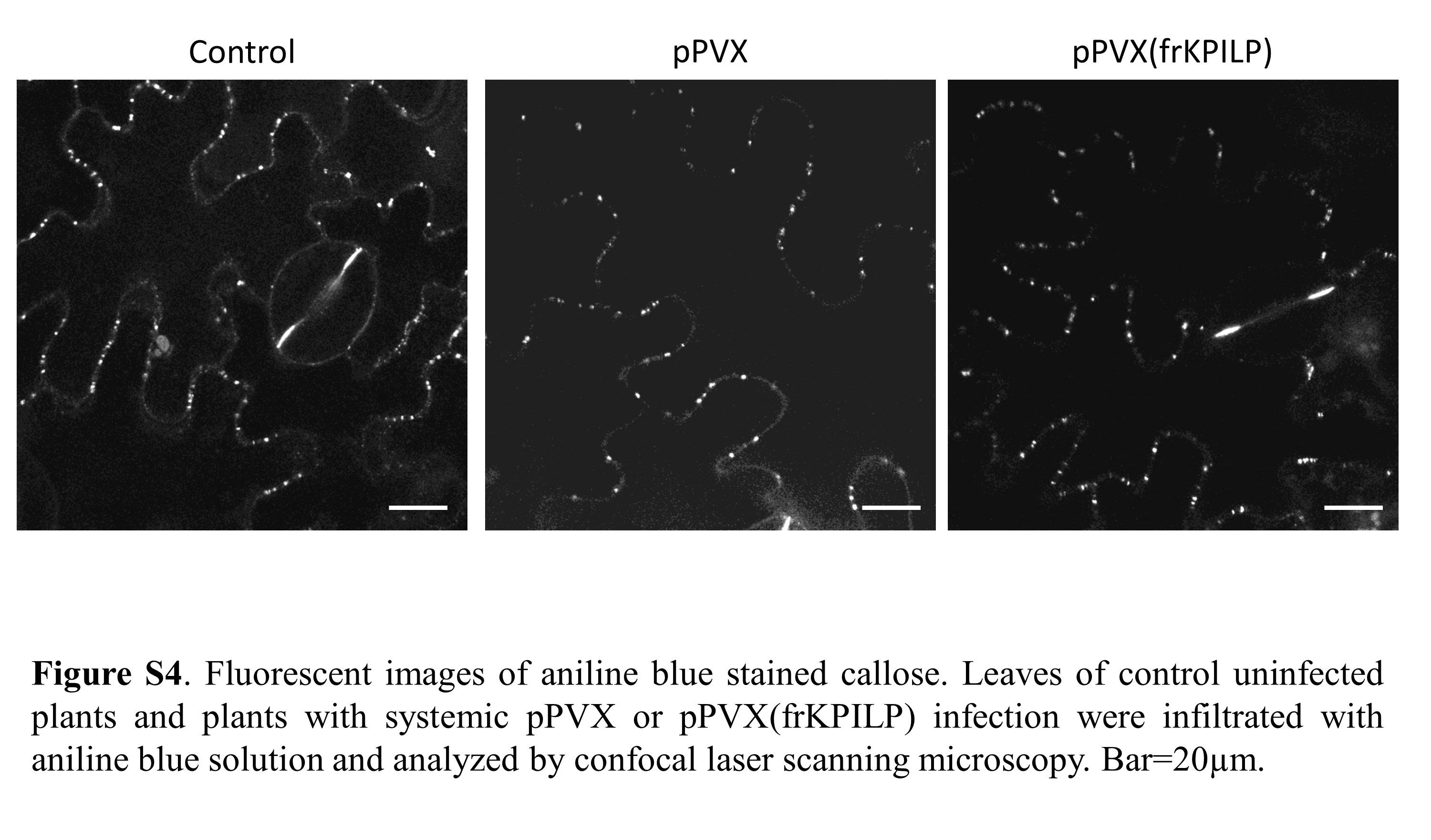

Supplement: Supplementary file 4 [file Image_4.tif]
