# Supplementary material for: Nicotiana benthamiana Kunitz peptidase inhibitor-like protein involved in chloroplast-to-nucleus regulatory pathway in plant-virus interaction
Source: Front Plant Sci. 2022 Nov 10;13:1041867. doi: 10.3389/fpls.2022.1041867 (PMC9685412; doi:10.3389/fpls.2022.1041867)
Supplement: Supplementary file 5 [file Table_1.docx]

**Table S1. Oligonucleotides used for cloning**

| Oligonucleotide name | Sequence |
| --- | --- |
| F1 | GGTACCATGAAGATCATATCAAGGA |
| R1 | GTCGACTTAAACCTTCTTGAACACAAT |
| F2 | TCGCGAACCAGTTCTTGATAC |
| R2 | GTCGACATTAAGAATTTTAAAGGAAG |
| F8 | GAAAGGCGAAGCCGGGAGCTGC |
| R8 | GCAGCTCCCGGCTTCGCCTTTC |
| R9 | CCTGCACTTGAAGCCACCATTAAG |
| F9 | CTTAATGGTGGCTTCAAGTGCAGG |
| R10 | CAAACTTGTGGCCACCCCGTTTTCTG |
| F10 | GGTGGCCACAAGTTTGTTTAC |
