# Supplementary material for: Nicotiana benthamiana Kunitz peptidase inhibitor-like protein involved in chloroplast-to-nucleus regulatory pathway in plant-virus interaction
Source: Front Plant Sci. 2022 Nov 10;13:1041867. doi: 10.3389/fpls.2022.1041867 (PMC9685412; doi:10.3389/fpls.2022.1041867)
Supplement: Supplementary file 6 [file Table_2.docx]

**Table S2**. **Oligonucleotides used for qRT-PCR**

| Gene | Forward primer | Reverse primer | Accession number |
| --- | --- | --- | --- |
| *18S rRNA* | ACGGCTACCACATCCAAG | ACTCATTCCAATTACCAGACTC | KP824745.1* |
| *PP2A* | ATTGCTGCCTGTGGTTATTAC | ATAGACTGAAGTGCTTGATTGG | MF996339.1* |
| *KPILP* | TGAGCACTGGCGGAATTAAGG | ATACCAATATACCCACACAACAATCTG | FN687760.1* |
| *Pol PVX* | CTAGGTCTACCCAAGATTA | GTGGTCTCACAGTTTATG | MF405302.1* |
| *PsbQ* | AGCCGTAGAGCCGTCATC | GAGTTCAAAGTTCCAGGTAATCC | AY887536.1* |
| *GLK* | GTCTCACTCGCCATAACATAGC | TTTCCGCCACCTCCAACC | Niben101Scf06721g00011.1^#^ |
| *LHCB1* | TGTCGCCAAACCCGTCGCATCT | TTCACCGGTCAAGTAACTTGGG | Niben101Scf03455g01031.1^#^ |
| *LHCB2* | TGCGACGTACGGTTAGAAGT | CTCGGAGAATGGTCCCAAGT | Niben101Scf01987g00015.1^#^ |
| *RBCS1A* | GCTGCCTCATTCCCTGTTTC | CCTGCATGCATTGCACTCTT | Niben101Scf03015g06015.1^#^ |
| *HEMA1* | ATGTGGGTGCTTGTGTGAAC | AGGCGGTCCTCCTTATTAGC | Niben101Scf03068g00024.1^#^ |
| *XTH5* | ATCTTTGTGGATGACGTCCC | CATCTGCATCCCAAAGGCTC | Niben101Scf00369g05011.1^#^ |

*GeneBank database https://www.ncbi.nlm.nih.gov/genbank

^#^SolGenomics database [https://solgenomics.net](https://solgenomics.net/)
